# Directed natural product biosynthesis gene cluster capture and expression in the model bacterium *Bacillus subtilis*

**DOI:** 10.1038/srep09383

**Published:** 2015-03-24

**Authors:** Yongxin Li, Zhongrui Li, Kazuya Yamanaka, Ying Xu, Weipeng Zhang, Hera Vlamakis, Roberto Kolter, Bradley S. Moore, Pei-Yuan Qian

**Affiliations:** 1 KAUST Global Collaborative Research, Division of Life Science, Hong Kong University of Science and Technology, Clear Water Bay, Hong Kong; 2 Center for Marine Biotechnology and Biomedicine, Scripps Institution of Oceanography, University of California at San Diego, La Jolla, CA 92093, United States; 3 JNC Corporation, Yokohama Research Center, 5-1 Okawa, Kanazawa-ku, Yokohama, Kanagawa 2368605, Japan; 4 Department of Microbiology and Immunobiology, Harvard Medical School, Boston, MA 02115, United States; 5 Skaggs School of Pharmacy and Pharmaceutical Sciences, University of California at San Diego, La Jolla, CA 92093, United States

## Abstract

Bacilli are ubiquitous low G+C environmental Gram-positive bacteria that produce a wide assortment of specialized small molecules. Although their natural product biosynthetic potential is high, robust molecular tools to support the heterologous expression of large biosynthetic gene clusters in *Bacillus* hosts are rare. Herein we adapt transformation-associated recombination (TAR) in yeast to design a single genomic capture and expression vector for antibiotic production in *Bacillus subtilis*. After validating this direct cloning “plug-and-play” approach with surfactin, we genetically interrogated amicoumacin biosynthetic gene cluster from the marine isolate *Bacillus subtilis* 1779. Its heterologous expression allowed us to explore an unusual maturation process involving the *N*-acyl-asparagine pro-drug intermediates preamicoumacins, which are hydrolyzed by the asparagine-specific peptidase into the active component amicoumacin A. This work represents the first direct cloning based heterologous expression of natural products in the model organism *B. subtilis* and paves the way to the development of future genome mining efforts in this genus.

Next generation sequencing and genome mining technologies have revolutionized the discovery of natural product chemicals and biosynthetic enzymes that help fuel the fields of biotechnology and biomedicine^1^. Based on a recent comprehensive survey of publically available bacterial genomes, three phyla account for the majority of natural product biosynthetic potential, namely Actinobacteria, Proteobacteria, and Firmicutes^2^. While sophisticated molecular biology techniques have been firmly established to connect biosynthetic gene clusters to encoded natural product molecules in Actinobacteria and Proteobacteria through the use of model expression hosts such as *Streptomyces coelicolor* and *Escherichia coli*, Firmicutes, which contain the natural product rich genus *Bacillus*, are lacking comparable molecular tools to support the heterologous expression of large natural product pathways^3^,^4^. Here we report the design and implementation of a versatile vector to support the direct capture of *Bacillus* biosynthetic gene clusters from genomic DNA by transformation-associated recombination (TAR)^5^ in yeast and heterologous expression in the model host *Bacillus subtilis*.

*Bacillus subtilis* is a low G+C, Gram-positive bacterium that has been commonly used for decades in genetic and biochemical studies of chromosome replication and bacterial sporulation^6^,^7^. This species is an attractive option for the heterologous production of natural products for three main reasons. First, the *Bacillus* genus produces a wide assortment of biologically active small molecules, including antibacterial non-ribosomal cyclic lipopeptides of the surfactin and gageotetrin families, polyketides such as macrolactin and bacillaene, antitumor polyketide-peptide hybrids like amicoumacin and ieodoglucomide, and the discoipyrrole alkaloids (Fig. 1; Fig. S1)^8^,^9^,^10^,^11^,^12^. Second, *B. subtilis* has the capacity for natural genetic competence and subsequent homologous recombination, allowing the introduction of foreign DNA^13^,^14^. This feature offers a wide range of available genetic manipulation techniques to facilitate practical biosynthetic efforts of natural products. Indeed, the natural transformation system of *B. subtilis* is so effective that the 3.5-megabase genome of *Synechocystis* PCC6803 was successfully assembled into the *B. subtilis* 168 genome, which served as a cloning vector (also known as the *Bacillus* Genome (BGM) vector)^14^,^15^. This BGM cloning system has been applied to demonstrate the cloning of the entire mouse mitochondrion and rice chloroplast genomes^16^. And third, *B. subtilis* is non-pathogenic and is generally recognized as a safe production host that can satisfy safety requirements for the industrial productions of drug leads and enzymes^17^,^18^.

Despite these technical advantages and that *B. subtilis* is routinely used for protein expression, there are few reports utilizing *B. subtilis* as an expression host system for the production of natural product small molecules. Most reports have used chromosomal transfer or cosmid library expression techniques and have focused on relatively small pathways of ribosomal and non-ribosomal peptide products^19^,^20^,^21^,^22^,^23^,^24^,^25^,^26^,^27^*.* However, the limitations of gene cluster cloning via clone libraries^4^ and chromosomal transfer hinder the efficient study of gene clusters from undomesticated producers or large pathways. Furthermore, a critical bottleneck in using *B. subtilis* as a heterologous host is the lack of autonomous plasmids to facilitate cloning, transfer and heterologous expression of large biosynthetic gene clusters^3^.

We recently developed a new genetic platform for the efficient capture of a silent 67-kb biosynthetic gene clusters directly from genomic DNA via TAR in yeast to support a “plug-and-play” approach to small molecule production^28^. To date we have captured and expressed high G+C Gram-positive actinomycete pathways for the marinopyrrole taromycin and enterocin^28^,^29^ antibiotics and the Gram-negative pseudoalteromonad pathway for the alterochromides^30^. Herein we adapted this platform to support the capture and expression of low G+C Gram-positive bacilli-based natural products. We validated the method with the prototype *Bacillus* lipopeptide surfactins and the hybrid polyketide-peptide amicoumacins, which exhibit broad bioactivities, including antibacterial, antifungal and antitumor activities^12^,^31^,^32^,^33^. This work represents a very useful approach to interrogate the function of biosynthetic gene clusters in *Bacillus* through heterologous biosynthesis.

## Results

### Design and validation of the pCAPB gene cluster capture vectors

The gene cluster capture vector pCAP01 consists of three elements that allow direct capture and manipulation in yeast, maintenance and manipulation in *Escherichia coli*, and chromosomal integration and expression of cloned pathways in actinomycetes^28^. To repurpose this vector for *Bacillus* expression, we first replaced the actinomycete elements with the *Bacillus* element from pBU4^34^ to generate the yeast*/E. coli* shuttle-*B. subtilis* capture vector pCAPB1 in order to support conjugal transfer into various *Bacillus* species (Fig. S2). We evaluated this replication plasmid pCAPB1 with the prototype *Bacillus* lipopeptide surfactin, which is encoded on the 38-kb *srf* locus from *B. subtilis* 1779. Although we successfully captured the *srf* locus from genomic DNA via TAR and constructed the pCAPB1-*srf* vector (Fig. 2, Fig. S3), upon its transfer into five *Bacillus* host strains, the vector was not stable and did not allow for surfactin production.

To overcome the instability issue of pCAPB1, we next designed pCAPB2 based on the *amyE* chromosomal integration plasmid pDR111, which is used in heterologous protein expression experiments in *B. subtilis*^35^,^36^. We incorporated the yeast elements from pCAP01 into a derivative of pDR111 to give the yeast*/E. coli* shuttle-*B. subtilis* chromosome integrative capture vector pCAPB2 (Fig. 2). This plasmid is maintained as a single copy in yeast cells to avoid unintended multiple recombination events during TAR, while it functions at multiple copies in *E. coli* to provide sufficient plasmid DNA materials for transfer to *B. subtilis*. The vector was designed to allow specific integration of cloned gene clusters into the chromosome of *B. subtilis* JH642 via double crossover recombination into the *amyE* gene. We validated the function of pCAPB2 by direct cloning of the *srf* gene cluster from *B. subtilis* 1779 to give the integration plasmid pCAPB2-*srf* (Fig. 2, Fig. S3). Upon its introduction into *B. subtilis* ROM77, in which the native *srf* locus was disrupted (JH642, *srfAA::cat*)^37^, we clearly detected surfactin production at wild-type levels by UPLC-MS (Fig. S4).

### Identification and TAR capture of amicoumacin biosynthesis (*ami*) genes

**Figure 1:**
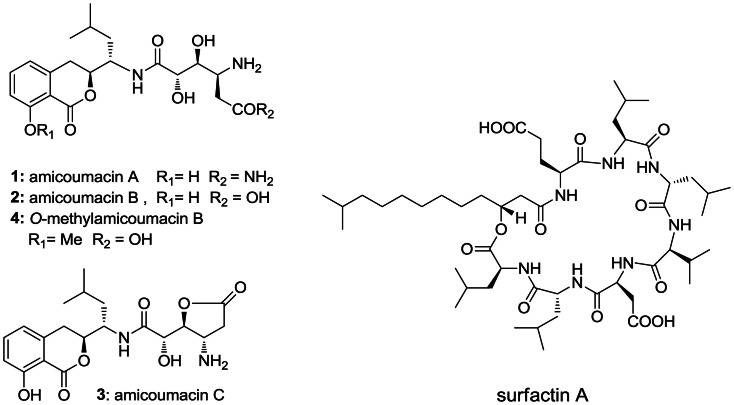
Chemical structure of amicoumacins (1–4) and surfactin.

**Figure 2:**
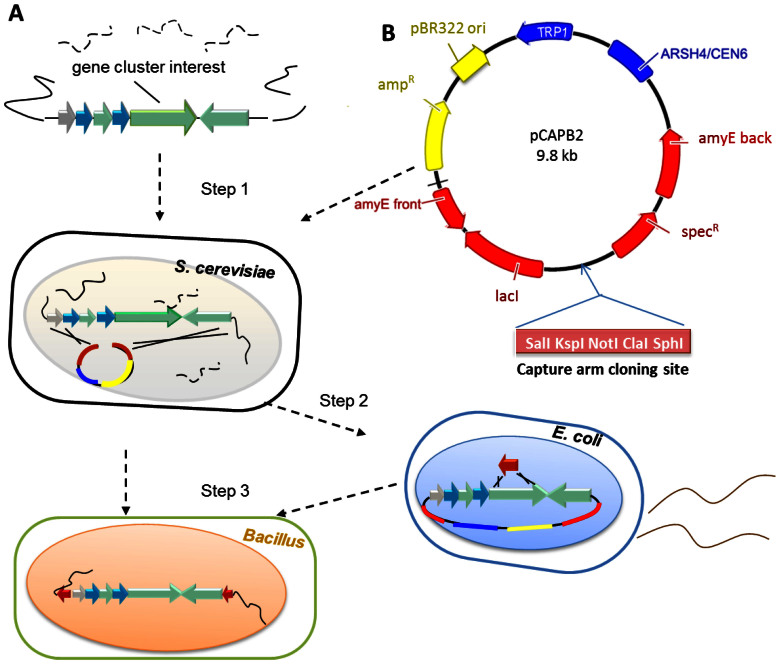
Design and strategy of TAR-based cloning and expression. (A) The procedure for TAR-based natural product heterologous expression in *Bacillus* involves three steps. In step 1, TAR in yeast involves homologous recombination between the linearized pathway specific capture vector and genomic DNA fragments to yield a circular construct that can form visible yeast colonies on selective media. In step 2, the cloned pathway can be manipulated using λ-Red recombination-mediated PCR targeting in *E. coli*. Finally, in step 3, through natural competence transformation, the cloned and manipulated pathway is integrated into the chromosome of *Bacillus subtilis* JH642 for natural products expression studies. (B) Physical map of the gene cluster capture vector pCAPB2 used in TAR direct cloning. The vector consists of three elements that allow direct cloning of pathways in yeast (blue), maintenance and manipulation in *E. coli* (yellow), and chromosomal integration and expression of cloned pathways in *B. subtilis* (red). The yeast element consists of ARSH4/CEN6 (replication origin) and TRP1 auxotrophic marker, while the *E. coli* and the *Bacillus* elements consist of DNA sequence for integration into the *B. subtilis amyE* gene, the *lac* repressor *lacI*,a spectinomycin resistance gene (*spec^R^*) for *Bacillus* and an ampicillin resistance gene for *E. coli* (*amp^R^*). For the construction of a pathway specific capture vector, homology arms corresponding to both ends of the pathway are introduced into the capture arm cloning sites.

With the successful completion of the proof-of-principle experiment to directly capture and express a *Bacillus* natural product pathway in *B. subtilis*, we next turned our attention to an uncharacterized *Bacillus* pathway to further showcase the proficiency of the pCAPB2 system. We selected to evaluate the amicoumacins, which are bioactive isocoumarin natural products that were first reported from *Bacillus pumilus* in 1981^31^. Recent biochemical studies revealed that amicoumacin belongs to a new class of protein synthesis inhibitors that binds to the ribosome^38^,^39^. We re-isolated the amicoumacins from the Red Sea isolate *B. subtilis* 1779 following a bioassay-guided isolation procedure to give amicoumacins A-C (**1**-**3**) and *O*-methylamicoumacin B (**4**) (Fig. 1), based on high-resolution MS and NMR characterization. We suspect that some of the amicoumacin analogues may be byproducts generated from amicoumacin A (**1**) during the isolation and sample preparation processes. To capture and express the amicoumacin (*ami*) gene cluster, which we hypothesized to be encoded by a hybrid modular polyketide synthase-nonribosomal peptide synthetase (PKS-NRPS), we sequenced the wild-type producer *B. subtilis* 1779 genome.

**Figure 3:**
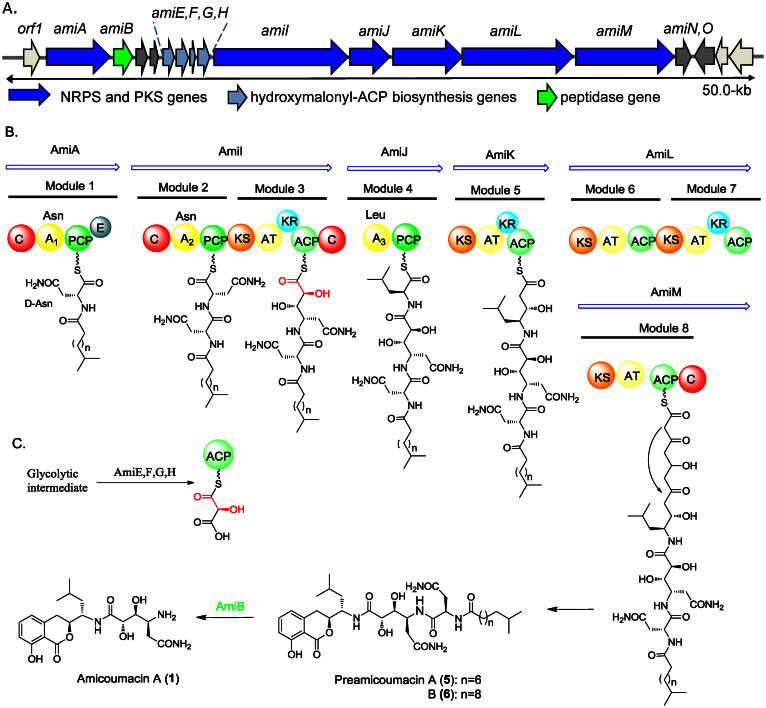
Proposed biosynthetic pathway of amicoumacins. (A) Organization of the *ami* biosynthetic gene cluster in *Bacillus subtilis* 1779; (B) Proposed biosynthetic route to amicoumacins in *B. subtilis* 1779; (C) Postulated biosynthetic steps leading to hydroxymalonyl-ACP, which is a proposed PKS extender unit of the module 3. Domain abbreviations: A, adenylation; ACP, acyl carrier protein; AT, acyltransferase; C, condensation; E, epimerase; KR, ketoreductase; KS, ketosynthase; PCP, peptidyl carrier protein.

Sequence analysis revealed several assembly line biosynthetic gene clusters, including a contiguous region of 47.4 kb containing 16 open reading frames (ORFs) that we predicted were responsible for amicoumacin biosynthesis (Fig. 3A, Table S3). We designated these genes *amiA-O.* Inspection of this locus identified an NRPS-PKS hybrid protein encoded by *amiI,* two NRPSs encoded by *amiA* and *amiJ*, and three PKS genes encoded by *amiK-M.* The predicted biosynthesis gene cluster consists of eight modules in total, as shown in Fig. 3, for the incorporation of three amino acids and five malonate residues. Based on the co-linearity rule of assembly line biosynthesis, we suspected that the predicted initiation module encoded by *amiA* synthesizes a fatty acyl-D-Asn residue reminiscent of lipopeptide natural products, suggesting that the product of the AmiA-M megasynthetase may be a derivative of lipoamicoumacins A–D that we previously reported^40^. We thus explored the possibility that the immediate product of the *ami* biosynthetic pathway may not be amicoumacin A but rather a lipidated precursor that may support a pro-drug-like activation mechanism (Fig. 3). The unusual dihydroisocoumarin core structure is likely formed by the terminating AmiJ–M megasynthetase proteins to generate a highly oxygenated polyketide chain that rearranges into the bicyclic dihydroisocoumarin moiety. Feeding experiments with ^15^N_2_-L-asparagine and 5,5,5-trifluoro-DL-leucine followed by MS analyses further supported the proposed amicoumacin A biosynthetic pathway (Table S4).

**Figure 4:**
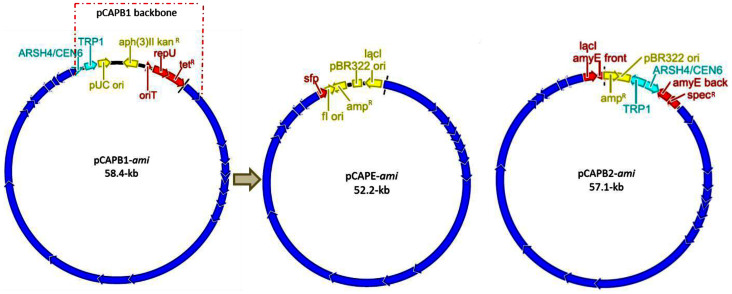
Physical maps of the TAR-cloned *ami* gene cluster and heterologous expression vectors. The 47.4-kb genomic region containing the *ami* gene cluster (blue) was directly cloned in yeast, yielding pCAPB1-*ami* and pCAPB2-*ami*. The pCAPB1 backbone and unrelated gene *orf*1 on pCAPB1-*ami* (dotted arrows) were replaced with the *ami* specific capture vector pCAPE in *E. coli* cells via λ-red mediated recombination, generating *ami* gene cluster *E. coli* expression construct pCAPE-*ami*.

Owing to the projected unusual natural product activation mechanism, we initially interrogated the biosynthetic pathway of amicoumacins within the wild-type producer strain *B. subtilis* 1779. However, all of our attempts to disrupt target genes in the native strain were unsuccessful, as had been other attempts to previously analyze amicoumacin production^41^. Therefore, to study the amicoumacin biosynthesis pathway, we targeted the genomic region containing the 47.4-kb *ami* gene cluster for TAR and heterologous expression in either *Bacillus subtilis* or *E. coli*. We used 1-kb capture arms corresponding to the periphery of the *ami* locus to generate an *ami* pathway specific capture vector in pCAPB2. *Saccharomyces cerevisiae* VL6-48 was transformed with the linearized capture vector and genomic DNA fragments of *B. subtilis* 1779. Positive clones were identified by PCR and transferred to *E. coli* for propagation to give the heterologous expression construct pCAPB2-*ami* (Fig. 4, Fig. S5).

### Heterologous expression of the amicoumacin biosynthesis genes

**Figure 5:**
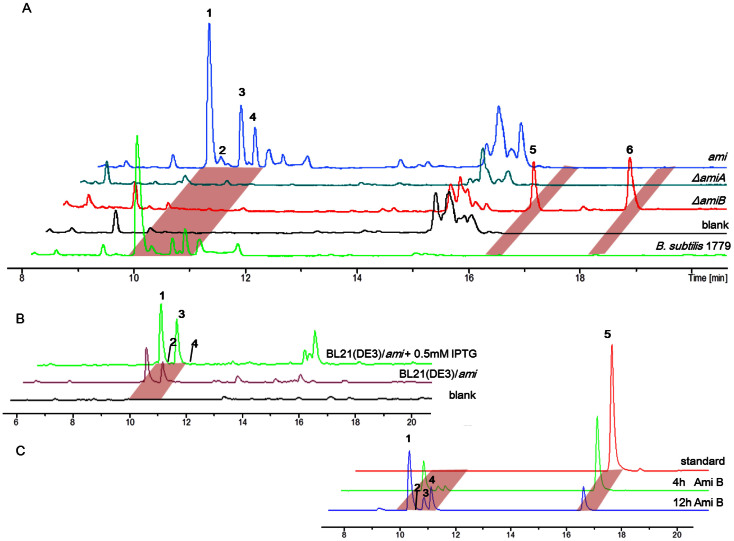
UPLC-MS analyses of heterologously produced amicoumacins*.* (A) LCMS UV traces showing the relative production of amicoumacins (**1**–**6**) in the native *B. subtilis* 1779 and *B. subtilis* JH642+*sfp* carrying heterologous expression constructs pCAPB2-*ami*, pCAPB2-*ami* (Δ*amiA*), pCAPB2-*ami* (Δ*amiB*), and pCAPB2 (blank) with UV monitoring at 314 nm. (B) LCMS extracted ion chromatogram traces of amicoumacins (**1**-**6**) produced by *E. coli* BL21(DE3) carrying pCAPE-*ami* (0, 0.5 mM IPTG) and the empty vector pCAPE. (C) LCMS extracted ion chromatogram traces of compounds (**1**-**5**) showing the *in vivo* conversion of preamicoumacin A (**5**, “standard”) to amicoumacins (**1**–**4**) after 4 and 12 hours incubations in *B. subtilis* JH642+*amiB*.

For the heterologous expression of the *ami* gene cluster, we introduced the integration plasmid pCAPB2-*ami* into the genome of *B. subtilis* JH642+*sfp* in which the phosphopantetheinyl transferase gene *sfp* has been added^42^. The resultant transformants successfully produced amicoumacins **1**–**4** at comparative levels to that in *B. subtilis* 1779, as revealed by UPLC-MS (Fig. 5A) and NMR analyses. We additionally expressed the *ami* cluster in *E. coli* BL21(DE3) via the construction of a different capture vector based on pETDuet-1, which also resulted in amicoumacin production, albeit at levels 100-fold less than that in the native *B. subtilis* 1779 strain or in the *B. subtilis* JH642 host (Fig. 5B, Fig. S6).

The heterologous production of the amicoumacins in the *B. subtilis* and *E. coli* hosts provided unequivocal evidence that the *ami* locus encodes amicoumacin biosynthesis. With these systems in hand, we further interrogated the function of *amiA* by λ-Red recombination-mediated PCR targeting in *E. coli* BW25113^43^. Restriction mapping of the plasmid propagated in *E. coli* confirmed that the *amiA* gene was successfully replaced by a gene encoding apramycin resistance to yield pCAPB2-*ami* (Δ*amiA*) (Fig. S5). This construct was integrated into the chromosome of the *B. subtilis* host, whereupon we observed that amicoumacin production was now lost (Fig. 5A).

### Pro-drug mechanism of amicoumacin activation

With the successful heterologous expression of the amicoumacin biosynthetic gene cluster and the ability to readily inactivate individual *ami* genes, we next explored the molecular and functional relationship between the various structures and the possibility of a pro-drug-like activation strategy. To this end, we first examined *amiB*, which codes for a D-Asn peptidase homologous to XcnG and ClbP that convert inactive precursors into the antibiotics xenocoumacin and colibactin, respectively^44^,^45^,^46^. To evaluate if AmiB is similarly involved in activating lipoamicoumacin-like precursors to form mature amicoumacin antibiotics, we mutated the *amiB* gene in the pCAPB2-*ami* plasmid (Fig. S5) and expressed pCAPB2-*ami* (Δ*amiB*) in the *B. subtilis* host. The mutant strain lacking *amiB* was then analyzed by UPLC-MS analysis (Fig. 5A). Indeed, as predicted, new shunt products were produced instead of amicoumacins in the *amiB* deletion mutant. These products were isolated and characterized as preamicoumacins A–B (**5**–**6**) (Fig. 3B) by comprehensive NMR, MS, and Marfey analyses (Table S5, Fig. S7–S8, and Supplemental Experimental Procedures). Preamicoumacin A resembles lipoamicoumacin A and specifically differs from amicoumacin A by tailoring the amine group at C-10′ with *N*-acyl Asn as predicted bioinformatically. The configuration of the *N*-acyl Asn residue was assigned as D on the basis of the advanced Marfey’s methods^47^,^48^. Both **5** and **6** represent derivatives of amicoumacin A extended at the *N* terminus by D-Asn carrying two different acyl chains (Fig. 3B).

With the structures of preamicoumacins A–B in hand, we were able to investigate the biochemical function of the AmiB, which is predicted to be a membrane-associated peptidase. To directly observe the cleavage of preamicoumacins to amicoumacin A, we heterologously expressed AmiB in *B. subtilis* JH642 for *in vivo* tests of proteolytic activity against preamicoumacins. Addition of exogenous **5** to *B. subtilis* carrying the gene *amiB* resulted in its conversion to **1** (Fig. 5C). While amicoumacin A (**1**) is active against *Staphylococcus aureus* (UST950701-005) with an MIC of 5.0 µg mL^−1^, derivatives **5** and **6** were inactive (Table S6). These results support the biosynthetic scenario whereupon inactive preamicoumacin precursors are first synthesized and then converted to the active component amicoumacin A.

## Discussion

Connecting genes to molecules with the help of efficient heterologous expression techniques is beginning to fundamentally change the natural product discovery paradigm. Herein we add the *Bacillus* antimicrobial compounds surfactin and amicoumacins to the small yet growing list of TAR captured and heterologously expressed microbial compounds^28^,^29^,^30^,^49^,^50^, thereby opening up the metabolically rich *Bacillus* genus to future natural product discovery efforts. In the present study, the TAR-directed capturing of the amicoumacin biosynthesis gene cluster from a marine *B. subtilis* isolate allowed for its heterologous expression and biosynthetic interrogation in a host *B. subtilis* strain, which represents a common genetic procedure practiced in other bacterial systems but not before with *Bacillus*. Our mutational work allowed us to establish an antibiotic “pro-drug” activation pathway in which newly discovered preamicoumacins are converted by the AmiB peptidase into the biologically active isocoumarin antibiotic in a process resembling xenocoumacin and colibactin processing in Gram-negative bacteria^44^,^46^ but not before observed in Gram-negative bacteria. This study was greatly facilitated with the pCAPB2 expression system that should similarly support the discovery and characterization of new chemical entities and enzymatic processes in other bacilli, which is an active pursuit of our laboratories.

## Methods

### Strains, fermentation, and isolation of amicoumacin compounds

All strains, plasmids and oligonucleotides used in this study are listed in Table S1–S2. *Bacillus subtilis* 1779 was isolated from seawater collected from the Red Sea during a 2010 research cruise. Its crude extract showed strong antibacterial activity against *S. aureus* UST950701-005. Detailed culture conditions and isolation procedures of amicoumacins A–C and *O*-methylamicoumacin B (**1**–**4**) are described in the Supplemental Experimental Procedures. For identification of preamicoumacins A–B, *B. subtilis* JH642+*sfp* that carried pCABP1-*ami* (Δ*amiB*) was cultured in LB media for 24 hours, extracted with ethyl acetate, and isolated by preparative RP-HPLC (60–100% MeCN in 0.1% trifluoroacetic acid, 40 min gradient) to obtain pure compounds. Analytical details of the preamicoumacins are provided in the Supplemental Information.

### Genome sequencing, annotation, and bioinformatics analysis of the *ami* gene cluster

The draft genome of *B. subtilis* 1779 was sequenced on an Illumina Hiseq2000 to generate 490-fold coverage of the 4.25 Mb genome. A total of 10.5 million pairs of Illumina reads were obtained from a 200 bp paired-end library. Genome assembly was performed with Velvet 1.0.15 with the following custom parameters: hash-length = 55 and coverage cutoff = 30. The draft genome sequences were deposited in GenBank as accession number SRS606572. The bioinformatics program antiSMASH (http://antismash.secondarymetabolites.org/)^51^ was initially used to analyze the whole draft genome sequence. The sequence of the orphan 47.4-kb NRPS/PKS hybrid gene cluster *amiA-O* encoded on contig-29 (95,452–140,246 nt) was further predicted and annotated using Pfam analyses (http://pfam.sanger.ac.uk/) and protein-protein BLAST (http://blast.ncbi.nlm.nih.gov). NRPS A domain specificities were analyzed using online program NRPSpredictor2 (http://nrps.informatik.uni-tuebingen.de/Controller?cmd=SubmitJob)^51^,^52^.

### Construction of the gene cluster capture vectors pCAPB1 and pCAPB2

Our initial attempts of replication plasmid pCAPB1 generated from our previous capture vector for *Streptomyces* pCAP01 by replacing *Streptomyces* element with *Bacillus* element from plasmid pBU4 was not successful for heterologous expression in *B. subtilis*. To generate the integration vector pCAPB2, the yeast element consisting of ARSH4/CEN6 (replication origin) and TRP1 auxotrophic marker from pCAP01, the *E. coli* and the *Bacillus* elements consisting of DNA sequence for integration into the *B. subtilis amyE* gene, the *lac* repressor *lacI* and an IPTG-inducible promoter, a spectinomycin resistance gene (*spec^R^*) for *Bacillus* and an ampicillin resistance gene for *E. coli* (*amp^R^*) from the pDR111 were assembled in *E. coli* Top10. For capture vector of heterologous expression in *E. coli* BL21 (DE3), the phosphopantetheine transferase (PPTase) gene *sfp* was inserted into MCS2 of pETDuet-1 to generate capture vector pCAPE. Detailed information is provided in Supplemental Experimental Procedures.

### Direct cloning of the *ami* gene cluster using TAR

Producer strain *B. subtilis* 1779 was grown in LB liquid medium overnight and genomic DNA was isolated from stationary phase cells. Approximately, 20 μg of genomic DNA were digested with 400 U of ScaI or SpeI, which did not cut the *ami* or *srf* gene clusters, respectively, in an overnight reaction at 37°C. The *ami* pathway-specific capture vector was constructed by introducing two PCR-amplified 1-kb homology arms corresponding to upstream and downstream regions of the *ami* gene cluster (*orf1* and *orf3*) into capture vector pCAPB1 and pCAPB2 (Fig. S2). To capture the *ami* gene cluster, spheroplast cells of *S. cerevisiae* VL6-48 were transformed with the linearized *ami* pathway-specific capture vector and enzymatically fragmented genomic DNA together. Desired transformants with the captured *ami* gene cluster were selected on synthetic tryptophan dropout agar and identified by PCR. Direct cloning of the *ami* cluster was confirmed by restriction mapping to give pCAPB1-*ami* and pCAPB2-*ami*. The surfactin gene cluster *srf* was similarly captured following the same protocol to give pCAPB1-*srf* and pCAPB2-*srf.* For the *ami* gene cluter expression in *E. coli,* competent cells of *E. coli* BW25113 carrying pIJ790 and pCAPB1-*ami* were transformed with the linearized capture vector pCAPE to generate pCAPE-*ami* via λ-Red mediated recombination. The pCAPB1-*ami*, pCAPB2-*ami* and pCAPE-*ami* constructs were obtained and confirmed by restriction mapping after stable propagation through *E. coli* (Fig. 4, Fig. S5). More detailed information is provided in Supplemental Experimental Procedures.

### Heterologous expression of the *ami* gene cluster

The construct pCAPB2-*ami* and its derivatives, which have 1.0-kb homology regions corresponding to the upstream and downstream regions of the gene *amyE*, were transferred to strain *B. subtilis* JH642+*sfp* by natural competence transformation. Spectinomycin-resistant and PCR positive clones were routinely grown in LB broth containing spectinomycin (100 μg mL^−1^) at 30°C overnight. A portion (1.0 mL) of the preculture was inoculated into 100 mL of LB broth and grown for 1 d at 30°C in a 250-mL flask with rotary shaking. For heterologous expression in *E. coli*, construct pCAPE-*ami* was introduced into BL21 (DE3) cells via electroporation. The positive clones were inoculated and confirmed by restriction mapping and then similarly cultured. The EtOAc extracts from the culture broth were analyzed by reversed-phase UPLC-MS. Detailed information, including the analytical conditions for UPLC-MS, genetic manipulation of the genes *amiA* and *amiB*, sample preparation for UPLC-MS analysis, and antimicrobial bioassay, are described in Supplemental Information.

## Supplementary Material

Supplementary Information Directed natural product biosynthesis gene cluster capture and expression in the model bacterium Bacillus subtilis

## References

[b1] Bachmann B. O., Van Lanen S. G. & Baltz R. H. Microbial genome mining for accelerated natural products discovery: Is a renaissance in the making? J. Ind. Microbiol. Biotechnol. 41, 175–184 (2014). 24342967 10.1007/s10295-013-1389-9 PMC4070288

[b2] Cimermancic P. *et al.* Insights into secondary metabolism from a global analysis of prokaryotic biosynthetic gene clusters. Cell 158, 412–421 (2014). 25036635 10.1016/j.cell.2014.06.034 PMC4123684

[b3] Zhang H. R., Boghigian B. A., Armando J. & Pfeifer B. A. Methods and options for the heterologous production of complex natural products. Nat. Prod. Rep. 28, 125–151 (2011). 21060956 10.1039/c0np00037j PMC9896020

[b4] Ongley S. E., Bian X. Y., Neilan B. A. & Muller R. Recent advances in the heterologous expression of microbial natural product biosynthetic pathways. Nat. Prod. Rep. 30, 1121–1138 (2013). 23832108 10.1039/c3np70034h

[b5] Kouprina N. & Larionov V. Selective isolation of genomic loci from complex genomes by transformation-associated recombination cloning in the yeast *Saccharomyces cerevisiae*. Nat. Protoc. 3, 371–377 (2008). 18323808 10.1038/nprot.2008.5

[b6] Moszer I. *et al.* Subtilist: The reference database for the *Bacillus subtilis* genome. Nucleic Acids Res. 30, 62–65 (2002). 11752255 10.1093/nar/30.1.62 PMC99059

[b7] Kunst F. *et al.* The complete genome sequence of the gram-positive bacterium *Bacillus subtilis*. Nature 390, 249–256 (1997). 9384377 10.1038/36786

[b8] Tareq F. S. *et al.* Gageotetrins A-C, noncytotoxic antimicrobial linear lipopeptides from a marine bacterium *Bacillus subtilis*. Org. Lett. 16, 928–931 (2014). 24502521 10.1021/ol403657r

[b9] Tareq F. S. *et al.* Ieodoglucomides A and B from a marine-derived bacterium *Bacillus licheniformis*. Org. Lett. 14, 1464–1467 (2012). 22360451 10.1021/ol300202z

[b10] Stein T. *Bacillus subtilis* antibiotics: Structures, syntheses and specific functions. Mol. Microbiol. 56, 845–857 (2005). 15853875 10.1111/j.1365-2958.2005.04587.x

[b11] Hu Y. C. *et al.* Discoipyrroles A-D: Isolation, structure determination, and synthesis of potent migration inhibitors from *Bacillus hunanensis*. J. Am. Chem. Soc. 135, 13387–13392 (2013). 23984625 10.1021/ja403412y PMC3845659

[b12] Hamdache A., Lamarti A., Aleu J. & Collado I. G. Non-peptide metabolites from the genus *Bacillus*. J. Nat. Prod. 74, 893–899 (2011). 21401023 10.1021/np100853e

[b13] Dubnau D. Genetic competence in *Bacillus subtilis*. Microbiol. Rev. 55, 395–424 (1991). 1943994 10.1128/mr.55.3.395-424.1991 PMC372826

[b14] Itaya M., Tsuge K., Koizumi M. & Fujita K. Combining two genomes in one cell: Stable cloning of the *Synechocystis* PCC6803 genome in the *Bacillus subtilis* 168 genome. Proc. Natl. Acad. Sci. USA 102, 15971–15976 (2005). 16236728 10.1073/pnas.0503868102 PMC1276048

[b15] Itaya M. *et al.* Efficient cloning and engineering of giant DNAs in a novel *Bacillus subtilis* genome vector. J. Biochem. 128, 869–875 (2000). 11056400 10.1093/oxfordjournals.jbchem.a022825

[b16] Itaya M., Fujita K., Kuroki A. & Tsuge K. Bottom-up genome assembly using the *Bacillus subtilis* genome vector. Nat. Methods. 5, 41–43 (2008). 18066072 10.1038/nmeth1143

[b17] de Boer A. S. & Diderichsen B. On the safety of *Bacillus subtilis* and *B. amyloliquefaciens* - a review. Appl. Microbiol. Biotechnol. 36, 1–4 (1991). 1367772 10.1007/BF00164689

[b18] Leuschner R. G. K. *et al.* Qualified presumption of safety (QPS): A generic risk assessment approach for biological agents notified to the European food safety authority (EFSA). Trends. Food. Sci. Tech. 21, 425–435 (2010).

[b19] Borisova S. A. *et al.* Biosynthesis of rhizocticins, antifungal phosphonate oligopeptides produced by *Bacillus subtilis* ATCC6633. Chem. Biol. 17, 28–37 (2010). 20142038 10.1016/j.chembiol.2009.11.017 PMC2819989

[b20] Choi S. K. *et al.* Identification of a polymyxin synthetase gene cluster of *Paenibacillus polymyxa* and heterologous expression of the gene in *Bacillus subtilis*. J. Bacteriol. 191, 3350–3358 (2009). 19304848 10.1128/JB.01728-08 PMC2687177

[b21] Eppelmann K., Doekel S. & Marahiel M. A. Engineered biosynthesis of the peptide antibiotic bacitracin in the surrogate host *Bacillus subtilis*. J. Biol. Chem. 276, 34824–34831 (2001). 11448966 10.1074/jbc.M104456200

[b22] Herzner A. M. *et al.* Expression of the lantibiotic mersacidin in *Bacillus amyloliquefaciens* FZB42. PLoS One 6, e22389. (2011). 21811596 10.1371/journal.pone.0022389 PMC3141056

[b23] Liu W. & Hansen J. N. Conversion of *Bacillus subtilis* 168 to a subtilin producer by competence transformation. J. Bacteriol. 173, 7387–7390 (1991). 1938928 10.1128/jb.173.22.7387-7390.1991 PMC209249

[b24] Tsuge K. *et al.* Horizontal transfer of iturin a operon, *itu*, to *Bacillus subtilis* 168 and conversion into an iturin A producer. Antimicrob. Agents Chemother. 49, 4641–4648 (2005). 16251307 10.1128/AAC.49.11.4641-4648.2005 PMC1280175

[b25] Yuksel S. & Hansen J. N. Transfer of nisin gene cluster from *Lactococcus lactis* ATCC 11454 into the chromosome of *Bacillus subtilis* 168. Appl. Microbiol. Biotechnol. 74, 640–649 (2007). 17143619 10.1007/s00253-006-0713-y

[b26] Zobel S., Kumpfmuller J., Sussmuth R. D. & Schweder T. *Bacillus subtilis* as heterologous host for the secretory production of the non-ribosomal cyclodepsipeptide enniatin. Appl. Microbiol. Biotechnol. 99, 681–691 (2015). 25398283 10.1007/s00253-014-6199-0 PMC4306738

[b27] Tang Y. *et al.* Heterologous expression of an orphan nrps gene cluster from *Paenibacillus larvae* in *Escherichia coli* revealed production of sevadicin. J. Biotechnol. 194, 112–114 (2015). 25529345 10.1016/j.jbiotec.2014.12.008

[b28] Yamanaka K. *et al.* Direct cloning and refactoring of a silent lipopeptide biosynthetic gene cluster yields the antibiotic taromycin a. Proc. Natl. Acad. Sci. USA 111, 1957–1962 (2014). 24449899 10.1073/pnas.1319584111 PMC3918841

[b29] Bonet B. *et al.* Direct capture and heterologous expression of *Salinispora* natural product genes for the biosynthesis of enterocin. J. Nat. Prod. 10.1021/np500664q. (2014). PMC4380194 25382643

[b30] Ross A. C., Gulland L. E., Dorrestein P. C. & Moore B. S. Targeted capture and heterologous expression of the *Pseudoalteromonas* alterochromide gene cluster in *Escherichia coli* represents a promising natural product exploratory platform. ACS Synth. Biol., 10.1021/sb500280q. (2014) PMC4410906 25140825

[b31] Itoh J. *et al.* Amicoumacin-A, a new antibiotic with strong antiinflammatory and antiulcer activity. J Antibiot. (Tokyo) 34, 611–613 (1981). 7275843 10.7164/antibiotics.34.611

[b32] Peypoux F., Bonmatin J. M. & Wallach J. Recent trends in the biochemistry of surfactin. Appl. Microbiol. Biotechnol. 51, 553–563 (1999). 10390813 10.1007/s002530051432

[b33] Singh P. & Cameotra S. S. Potential applications of microbial surfactants in biomedical sciences. Trends Biotechnol. 22, 142–146 (2004). 15036865 10.1016/j.tibtech.2004.01.010

[b34] Bourgouin C., Delecluse A., Delatorre F. & Szulmajster J. Transfer of the toxin protein genes of *Bacillus sphaericus* into *Bacillus thuringiensis* subsp *israelensis* and their expression. Appl. Environ. Microbiol. 56, 340–344 (1990). 2306087 10.1128/aem.56.2.340-344.1990 PMC183341

[b35] van Ooij C. & Losick R. Subcellular localization of a small sporulation protein in *Bacillus subtilis*. J. Bacteriol. 185, 1391–1398 (2003). 12562810 10.1128/JB.185.4.1391-1398.2003 PMC142862

[b36] Wagner J. K., Marquis K. A. & Rudner D. Z. SirA enforces diploidy by inhibiting the replication initiator DNAa during spore formation in *Bacillus subtilis*. Mol. Microbiol. 73, 963–974 (2009). 19682252 10.1111/j.1365-2958.2009.06825.x PMC2992877

[b37] Solomon J. M., Magnuson R., Srivastava A. & Grossman A. D. Convergent sensing pathways mediate response to two extracellular competence factors in *Bacillus subtilis*. Genes. Dev. 9, 547–558 (1995). 7698645 10.1101/gad.9.5.547

[b38] Lama A. *et al.* Response of methicillin-resistant *Staphylococcus aureus* to amicoumacin A. *PLoS One*. 7, e34037 (2012). 10.1371/journal.pone.0034037 PMC3316591 22479511

[b39] Polikanov Y. S. *et al.* Amicoumacin A inhibits translation by stabilizing mRNA interaction with the ribosome. Mol. Cell 56, 531–540 (2014). 25306919 10.1016/j.molcel.2014.09.020 PMC4253140

[b40] Li Y. X. *et al.* Five new amicoumacins isolated from a marine-derived bacterium *Bacillus subtilis*. Mar. Drugs 10, 319–328 (2012). 22412803 10.3390/md10020319 PMC3296999

[b41] Pinchuk I. V. *et al.* Amicoumacin antibiotic production and genetic diversity of *Bacillus subtilis* strains isolated from different habitats. Res. Microbiol. 153, 269–276 (2002). 12160317 10.1016/s0923-2508(02)01320-7

[b42] Nakano M. M., Marahiel M. A. & Zuber P. Identification of a genetic-locus required for biosynthesis of the lipopeptide antibiotic surfactin in *Bacillus subtilis*. J. Bacteriol. 170, 5662–5668 (1988). 2848009 10.1128/jb.170.12.5662-5668.1988 PMC211666

[b43] Gust B. *et al.* PCR-targeted streptomyces gene replacement identifies a protein domain needed for biosynthesis of the sesquiterpene soil odor geosmin. Proc. Natl. Acad. Sci. USA 100, 1541–1546 (2003). 12563033 10.1073/pnas.0337542100 PMC149868

[b44] Bian X. Y. *et al.* In vivo evidence for a prodrug activation mechanism during colibactin maturation. Chembiochem 14, 1194–1197 (2013). 23744512 10.1002/cbic.201300208

[b45] Reimer D. & Bode H. B. A natural prodrug activation mechanism in the biosynthesis of nonribosomal peptides. Nat. Prod. Rep. 31, 154–159 (2014). 24356302 10.1039/c3np70081j

[b46] Reimer D. *et al.* A natural prodrug activation mechanism in nonribosomal peptide synthesis. Nat. Chem. Biol. 7, 888–890 (2011). 21926994 10.1038/nchembio.688

[b47] Fujii K. *et al.* A nonempirical method using LC/MS for determination of the absolute configuration of constituent amino acids in a peptide: Combination of Marfey's method with mass spectrometry and its practical application. Anal. Chem. 69, 5146–5151 (1997).

[b48] Fujii K. *et al.* Further application of advanced Marfey's method for determination of absolute configuration of primary amino compound. Tetrahedron Lett. 39, 2579–2582 (1998).

[b49] Kallifidas D. & Brady S. F. Reassembly of functionally intact environmental DNA-derived biosynthetic gene clusters. Methods Enzymol. 517, 225–239 (2012). 23084941 10.1016/B978-0-12-404634-4.00011-5 PMC3687798

[b50] Kim J. H. *et al.* Cloning large natural product gene clusters from the environment: Piecing environmental DNA gene clusters back together with TAR. Biopolymers 93, 833–844 (2010). 20577994 10.1002/bip.21450 PMC2895911

[b51] Blin K. *et al.* Antismash 2.0-a versatile platform for genome mining of secondary metabolite producers. Nucleic Acids Res. 41, W204–W212 (2013). 23737449 10.1093/nar/gkt449 PMC3692088

[b52] Rausch C. *et al.* Specificity prediction of adenylation domains in nonribosomal peptide synthetases (NRPS) using transductive support vector machines (TSVMS). Nucleic Acids Res. 33, 5799–5808 (2005). 16221976 10.1093/nar/gki885 PMC1253831

