# Supplementary material for: Directed natural product biosynthesis gene cluster capture and expression in the model bacterium Bacillus subtilis
Source: Sci Rep. 2015 Mar 24;5:9383. doi: 10.1038/srep09383 (PMC4894447; doi:10.1038/srep09383)
Supplement: Supplementary Information — Directed natural product biosynthesis gene cluster capture and expression in the model bacterium Bacillus subtilis [file srep09383-s1.doc]

**Directed natural product biosynthesis gene cluster capture and expression in the model bacterium *Bacillus subtilis***

Yongxin Li^1,2^, Zhongrui Li^1^, Kazuya Yamanaka^2,3^, Ying Xu^1^, Weipeng Zhang^1^, Hera Vlamakis^4^, Roberto Kolter^4^, Bradley S. Moore^2,5^* and Pei-Yuan Qian^1^*

^1^ KAUST Global Collaborative Research, Division of Life Science, Hong Kong University of Science and Technology, Clear Water Bay, Hong Kong

^2^ Center for Marine Biotechnology and Biomedicine, Scripps Institution of Oceanography, University of California at San Diego, La Jolla, CA 92093, United States

^3^JNC Corporation, Yokohama Research Center, 5-1 Okawa, Kanazawa-ku, Yokohama, Kanagawa 2368605, Japan

^4^ Department of Microbiology and Immunobiology, Harvard Medical School, Boston, MA 02115, United States

^5^ Skaggs School of Pharmacy and Pharmaceutical Sciences, University of California at San Diego, La Jolla, CA 92093, United States

* Author to whom correspondence should be addressed: boqianpy@ust.hk; bsmoore@ucsd.edu

1. **Experimental procedures**
2. **Strains, plasmids and oligonucleotides used in this study (Tables S1 and S2)**
3. **Deduced functions of the *ami* genes (Table S3)**
4. **Chemical structures of selected natural products from the genus *Bacillus*** **(Figure S1)**
5. **Physical maps of pCAPB1, pCAPB2, pCAPE, and their *srf* gene cluster derivatives (Figures S2 and S3)**
6. **Results of heterologous expression of the *srf* gene cluster in *Bacillus subtilis* ROM77 (Figure S4)**
7. **Restriction mappings of pCAPB1-*ami*, pCAPB2-*ami*, and pCAPE-*ami* (Figure S5).**
8. **Results of heterologous expression of the *ami* gene cluster in *Escherichia coli* BL21 (DE3) (Figure S6)**
9. **Results of feeding experiments for biosynthetic pathway study (Table S4)**
10. **NMR and MS analysis (Table S5, Figures S7, S8)**
11. **Antibacterial bioactivity of preamicoumacins (Table S6)**
12. **Efficiencies of conjugation between *E.coli* and *Bacillus* (Table S7)**
13. **Reference list for supplemental information**
14. **Annex: ESI-MS and NMR data of preamicoumacins A-B (5-6).**

**1. Experimental procedures**

**Strains and culture conditions**

Strains and plasmids, used in this study are listed in Tables S1 and S2. *B. subtilis* 1779 was isolated from marine sediment sample collected from the Red Sea during our 2010 research cruise. All *Bacillus* and *Escherichia coli* strains used in this study were routinely grown on solid Luria-Bertani (LB, pH 7.0) medium at 37°C and in liquid LB medium at 30°C with shaking at 180 rpm on a rotary shaker. For plasmid maintenance in *E. coli*, chloramphenicol (25 µg mL^-1^), ampicillin (100 µg mL^-1^) or kanamycin (40 µg mL^-1^) were used. *B. subtilis* recombinants were selected on LB medium containing spectinomycin (100 µg mL^-1^) or tetracycline (25 µg mL^-1^). The media for *Saccharomyces cerevisiae* strain VL6-48 (ATCC no. MYA-3666) which was used as a host for transformation associated recombination (TAR) direct cloning experiments [^1^](#_ENREF_1) were described in a previous study [^2^](#_ENREF_2).

**Constructions of the gene cluster capture vectors pCAPB1, pCAPB2, and pCAPE.**

All primers used in this study are listed in Table S2. To generate the capture vector pCAPB1 from our previous capture vector for *Streptomyces* pCAP01, the *Bacillus* element of *repU* and tetracycline-resistance marker gene was PCR amplified from self-replicable vector pBU4 (*Bourgouin et al., 1990*) with a pair of primers tet-F/R. The resultant product was digested with SphI and XhoI and was subsequently ligated with pCAP01 vector [^2^](#_ENREF_2) digested with the same enzymes, generating the yeast-*E. coli*-*Bacillus* shuttle capture vector pCAPB1. To generate the yeast-*E.coli*-*Bacillus* shuttle chromosome integrative capture vector pCAPB2, the yeast-element consisting of ARSH4/CEN6 (replication origin) and the TRP1 auxotrophic marker was introduced into pDR111, which carried a spectinomycin-resistance marker, a polylinker downstream of the P*_hyperspank_* promoter and the gene for the *lacI* repressor between two arms of the *amyE* gene (gifted from D. Rudner, Harvard Medical School) [^3^](#_ENREF_3) as follows. The yeast element was PCR amplified from pCAP01 with a pair of primers yeast-element-F/R, and the resultant product was digested and subsequently ligated into the SpeI and NcoI sites of pDR111, generating pCAPB2 vector. Additionally, the *sfp* gene flanked by NdeI and XhoI restriction sites was amplified by PCR from genomic DNA of *B. subtilis* 1779, and the resultant product was introduced into NdeI and XhoI sites in the second multiple cloning site of pETDuet-1, generating *E. coli* expression vector pCAPE.

**Construction of the *ami* and *srf* gene cluster specific capture vectors.**

The *ami* gene cluster specific capture vector was constructed on pCAPB1 by introducing two 1.0-kb capture arms corresponding to upstream and downstream peripheral regions of the *ami* gene cluster. As the upstream capture arm of the *ami* gene cluster, a 1.0-kb region corresponding to the *orf*1 was PCR amplified with a pair of primers ami1-Up-F/R, in which XhoI and BamHI restriction sites were introduced. Similarly, as downstream capture arm, the 1.0-kb region corresponding to *orf*2 was PCR amplified with primers ami1-down-F/R that harbored BamHI and KpnI restriction sites. The two PCR amplified arms were then assembled into single piece (2.0-kb) by overlap extension PCR with primers ami1-Up-F and ami1-Down-R. The assembled fragment was digested with XhoI and KpnI and introduced into equivalent sites of pCAPB1, yielding the *ami* gene cluster specific capture vector. The *ami* gene cluster specific capture vector on pCAPB2 was similarly generated with two pairs of primers ami2-Up-F/R and ami2-Down-F/R. For heterologous expression of the *ami* cluster in *E. coli,* capture arms were similarly introduced into the multiple cloning site 1 on pCAPE as follows. The capture arms corresponding to upstream and downstream regions of *ami* gene cluster were amplified and assembled by PCR with two pairs of primers ami*-*E-Up-F/R and ami*-*E-Down-F/R. The resultant product was digested and subsequently introduced into the NcoI and EcoRI restriction site of pCAPE, generating the third *ami* gene cluster specific capture vector. With the same procedure, the *srf* gene cluster specific capture vectors were also generated on pCAPB1 and pCAPB2 with four pairs of primers srf1-Up/Down-F/R and srf2-Up/Down-F/R, respectively. In prior to direct TAR cloning, the pathway specific capture vectors were digested with an appropriate restriction enzyme that cuts junction region of the two capture arms, yielding linear capture vectors.

**TAR direct cloning of the *ami* and *srf* gene clusters**

Genomic DNA from *B. subtilis* 1779 was extracted from overnight culture with standard protocol. Approximately 20 μg of genomic DNA were digested with 100 U of ScaI or SpeI, which do not cut the *ami* or *srf* gene clusters, respectively, in an overnight reaction at 37 °C. Direct TAR clonings of the *ami* and *srf* gene clusters from genomic DNA were carried out in highly transformable yeast *S. cerevisiae* strain VL6-48 according to a previously reported protocol [^2^](#_ENREF_2). Stabilized spheroplast cells were co-transformed with 0.4 to 1.2 μg of enzymatically digested genomic DNA fragments and 0.2 to 0.4 μg linearized gene cluster specific capture vector. Desired transformants were selected on synthetic tryptophan drop-out agar. For screening of the directly cloned *ami* and *srf* gene clusters, colony PCR was conducted using primers amplifying a 1.0-kb region in the middle of the *ami* and *srf* clusters, ami-check-F/R and srf-check-F/R, respectively. The identified constructs were extracted and propagated through *E. coli* transformation The yielded constructs were designated as pCAPB1-*ami*, pCAPB2-*ami*, pCAPB1-*srf*, and pCAPB2-*srf*. For *E. coli* expression, linear capture vector pCAPE with homology arms was replaced with pCAPB1 backbone and *orf1* on pCAPB1-ami by λ-red recombination in *E. coli* BW25113 / pIJ790 [^4^](#_ENREF_4). The resultant construct was designated as pCAPE-*ami*. The pCAPB1-*ami*, pCAPB2-*ami* and pCAPE-*ami* constructs were obtained and confirmed by confirmed by restriction analysis with BglII after stable propagation through *E. coli* (Figure S5).

**Genetic manipulation of the *ami* genes.**

Genetic manipulations were carried out using λ-Red recombination-mediated PCR targeting [^4^](#_ENREF_4). The *amiA* and *amiB* genes on pCAPB2-*ami* were individually replaced with the PCR amplified *aac(3)IV* (apramycin-resistance marker, apra^R^) gene flanked by 39 nucleotide homology arms as follows. The gene including its promoter was PCR amplified from plasmid pIJ773 with two pairs of primers amiA-Apra-F/R and amiB-Apra-F/R, respectively. The PCR product was then individually introduced into *E. coli* BW25113 cells carrying pIJ790 and pCAPB2-*ami* by electroporation. The resultant constructs were purified from apra^R^ clones and confirmed by restriction analysis with BglII, yielding pCAPB2-*ami* (Δ*amiA*) and pCAPB2-*ami* (Δ*amiB*) (Figure S5B).

**Introduction of the *ami* and *srf* gene clusters into heterologous *Bacillus* hosts.**

The self-replicable constructs pCAPB1-*ami*/*srf* were transferred to five *Bacillus* host strains including three *B. subtilis* strains (JH642+*sfp*, ROM77, and 168) and two *B. thuringiensis* strains (GBJ001 and BMB171) from *E. coli* ET12567 by triparental conjugal DNA transfer facilitated by *E. coli* ET12567 cells carrying pUB307[^7^](#_ENREF_7). The resultant exconjugants were selected on LB agar containing tetracycline (20 µg mL^-1^), and then plasmids were extracted to confirm successful DNA transfer. The conjugation efficiencies between *E. coli* and *B. subtilis* were showed in Table S6. Unfortunately, all plasmids extracted appeared not to harbor most part of the cloned gene cluster likely due to unintended recombination events in *Bacillus*. Thus, self-replicable plasmid carrying huge DNA fragment was realized to be unstable. To overcome the size issue, a new integrative capture vector pCAPB2 was used. pCAPB2-*ami* was introduced into chromosome of *B. subtilis* JH642+*sfp* through natural competence transformation [^8^](#_ENREF_8), while pCAPB2-*srf* was introduced into *B. subtilis* ROM77 (JH642, *srfAA::cat*). In order to check the stability of the integrated gene clusters, we monitored the encoded products and performed multiple PCR amplifications of several different regions in gene cluster after dozens rounds of cultures in LB medium with antibiotics. The target gene clusters maintained in hosts were stable and intact.

**Isolation of amicoumacin compounds.**

*Bacillus* strains including recombinants were cultivated in five 2.5 L flasks containing 1.0 L LB medium at 30 °C for 72 h. Chemical solvent EtOAc was added to the culture broth to extract their metabolites. The crude extract was separated by semi-preparative RP-HPLC column (Waters 600 apparatus using a semi-preparative C-18 Phenomenex Luna 5 μm (10 mm×250 mm) and monitored by a UV detector (Waters 2475)) with 50% MeCN in water to yield amicoumacins A-C (**1**-**3**) and *O*-methylamicoumacin B (**4**) at 1.5-3.2 mg L^−1^, respectively. For isolation of precursors preamicoumacins A-B (**5**-**6**), strain JH642+*sfp* carrying mutant construct pCAPB2-*ami* (Δ*ami*B) was cultivated in four 2.5 L flasks containing 1.0 L liquid LB medium and the compounds (**5**-**6**) (4.0, 3.6 mg) were purified from EtoAc extract of 4.0 L culture broth using semi-preparation RP-HPLC (60-100% MeOH, 40 min gradient).

**MS sample preparation and MS analysis of amicoumacins.**

For UPLC-ESI-MS analysis, metabolites from 10 mL of *Bacillus* cultures were extracted by EtOAc, and metabolites redissolved in 200ul MeOH were analyzed by Waters ACQUITY UPLC system (Waters ACQUITY, USA) coupled with a Bruker microTOF-q II mass spectrometer (Bruker Daltonics GmbH, Bremen, Germany). MS data were acquired in the positive ion mode with a range of 400-2000 m/z scans. Reversed-phase chromatography of UPLC was conducted with 2.1 x 150 mm columns (Waters, BEH C18, 1.7 µm, USA). HRESIMS spectra were recorded on a Bruker microTOF II ESI-TOF-MS spectrometer. The purified fractions of amicoumacins were also analyzed by UPLC-MS prior to NMR analysis.

**NMR analysis**

^1^H, ^1^H-^1^H-COSY, ^1^H-^13^C-HSQC, and ^1^H-^13^C-HMBC NMR spectra for compounds **1**-**6** were recorded on a Bruker AV500 spectrometer (500 MHz) using MeOH-*d*_4_ for **1**-**4** and DMSO-*d*_6_ for **5-6** (^1^H-NMR MeOH-*d_4_*: δ=3.31 ppm; DMSO-*d_6_*: δ=2.50 ppm; ^13^C-NMR: MeOH-*d_4_*: δ=49.00 ppm; DMSO-d_6_: δ=39.8 ppm). Preamicoumacin A (**5**) was obtained as a white amorphous solid. Based on HRESIMS (*m/z* 706.4028 [M+H]^+^, calc 706.4022) data, we established its molecular formula as C_35_H_55_N_5_O_10_. The UV spectrum was nearly identical to that reported for amicoumacins (λ_max_ 206, 247, and 314 nm), suggesting that **5** possessed a similar dihydroisocoumarin chromophore with that of amicoumacins (**1**-**4**) and lipoamicoumacins . The gross structure of **5** was further established by analyses of the ^1^H, ^13^C, ^1^H-^1^H COSY, HMQC, and HMBC NMR spectral data (Figure S7), indicating that its structure was closely related to that of lipoamicoumacin A [^10^](#_ENREF_10). The chemical shift of C-9’ and the ESI-MS spectrum showed fragment ions corresponding to the loss of a C11 acyl asparagine (Asn) (*m/z* 424), indicating that an amicoumacin A unit was linked to a acyl-Asn chain in preamicoumacin A instead of amicoumacin C unit in lipoamicoumacin A. These assignments were also supported by HMBC correlation from H-10' to C-14' and ESI-MS fragmentations (Figure S7, S8).

**Elucidation of amino acid configuration**

Amino acid configurations of preamicoumacins A-B (**5**-**6)** were determined using the advanced Marfey’s method . The compounds (**5**-**6**) (0.2 mg) were hydrolyzed in 6 M HCl at 110 °C overnight. Each solution was evaporated to dryness and the residue was dissolved in 100 μL water and divided into two portions. Each portion was treated with 20 μL NaHCO_3_ (1M) and 50 μL 1-fluoro-2, 4-dinitrophenyl-5-L-leucinamide (L-FDLA) or D-FDLA (1M) at 40 °C for 2 h. The reaction was quenched with 5 μL HCl (1M) and diluted with 200 μL MeOH. The stereochemistry was determined by comparison of the L-/D FDLA derivatized samples using UPLC-MS analysis (5.0% MeOH/H2O + 0.1% formic acid (FA) for 5 min followed by a gradient to 95% MeOH/H2O + 0.1% FA over 25 min at a flow of 0.25 mL min^-1^). Based on the hydroxylation of asparagine to aspartic acid, the elution order of the L/D-FDLA derivatized Asp residue (13.7/14.3 min) indicated that the Asn unit in preamicoumacins was D-Asn.

**Feeding experiments for biosynthetic pathway study**

Feeding experiments were performed with ^15^N_2_-L-asparagine and 5,5,5-trifluoro-DL-leucine in a synthetic medium [^13^](#_ENREF_13) that doesn’t contain L-asparagine and L-leucine, respectively. Prior to feeding experiment, *B. subtilis* 1779 was grown in LB medium at 30 °C with shaking at 180 rpm for overnight. Cells washed twice with the synthetic medium were then inoculated into 5 mL of the medium containing feeding precursors at a final concentration of 10 mM and was incubated at 30 °C with shaking at 180 rpm for 24 h. EtOAc extracts of the medium were analyzed in UPLC-MS.

**Heterologous expression and *in vivo* cleavage assay of AmiB**

The *amiB* gene was PCR amplified from the genomic DNA of *B. subtilis* 1779 using the primers amiB-HetEx-F/R. The resultant product was digested and cloned into HindIII and SphI sites of pDR111, generating pDR111-*amiB*. The purified plasmid pDR111-*ami*B from amp^R^ clone of *E. coli* was then transformed into *B. subtilis* via natural competence transformation for *in vivo* cleavage assay of peptidase AmiB. To study the cleavage of compounds **5** into **1** by AmiB, an *in vivo* assay in *B. subtilis* JH642 / *amiB* was performed as follows. *B. subtilis* JH642 / pDR111-*amiB* and *B. subtilis* JH642 / pDR111 (as the control) were cultivated in duplicates of 20 mL LB medium supplemented with spectinomycin (100 μg mL^-1^) in 100 mL flasks at 30 °C with shaking at 180 rpm on a rotary shaker. The experiments were started with inoculation of 20 ml of 4 h-old preculture (OD600 = 0.2) and administration of 0.1 mg of **5** dissolved in DMSO. Two mL of samples were taken at 4 h and12 h and extracted with 2 mL of EtOAc. The organic layer was evaporated to dryness and was redissolved in 50 μL of MeOH to analyze in UPLC-ESI-MS.

**Antibacterial assays**

The isolated compounds were evaluated by MIC assay against *B. subtilis* 1779 and *S. aureus* UST950701-005. Briefly, *B. subtilis* 1779 and *S. aureus* UST950701-005 were inoculated in liquid LB medium and grown at 30 °C for 12 h. The stock solution of samples were prepared at 25 mg mL^-1^ in DMSO and further diluted with LB medium and bacterial cultures to varying concentrations (100, 50, 20, 10, 5, 2, and 1 μg mL^-1^) in 96-well plates. The plates were incubated at 30°C for overnight. Cell growth was evaluated by measuring the optical density at 595 nm (Thermo scientific Multiskan FC multiplate photometer).

**2. Strains, plasmids and oligonucleotides used in this study (Tables S1 and S2)**

Table S1. Strains and plasmids used in this study.

| **Strain /Plasmid** | **Description** | **Source** |
| --- | --- | --- |
| **Strains** |  |  |
| *S. cerevisiae* VL6-48 | host strain for *in vivo* homologous recombination: MAT alpha, his3-D200, trp1-D1, ura3-52, lys2, ade2-101, met14, psi+cir0. | ATCC MYA-3666 |
| *E. coli* Top10 | host strain for routine cloning | Invitrogen |
| *E. coli* BL21(DE3) | host strain for routine heterologous expression | Invitrogen |
| *E. coli* BW25113 | K12 derivative: Δ*araBAD*, Δ*rhaBAD* | [^4^](#_ENREF_4) |
| *E. coli* ET12567 | DNA methylation deficient donor strain for conjugation | [^7^](#_ENREF_7) |
| *B. subtilis* 1779 | wild type producer strain of amicoumacins | this study |
| *B. subtilis* 168 | *trpC2, pheA1* | *Bacillus* Genetic Stock Center |
| *B. subtilis* ROM77 | JH642, *trpC2, pheA1, srfAA::cat* | [^14^](#_ENREF_14) |
| *B. subtilis* JH642+*sfp* | *trpC2, pheA1* +*sfp* | [^15^](#_ENREF_15) |
| *B. thuringiensis* GBJ001 | SmR mutant of *B. thuringiensis* serovar *israelensis* 4Q7, plasmid free | [^5^](#_ENREF_5) |
| *B. thuringiensis* BMB171 | Mutant of *B. thuringiensis* serovar *kurstaki* YBT-1463 | [^6^](#_ENREF_6) |
| **Plasmids** |  |  |
| pDR111 | *amyE::P_hyperspank_, lacI, spec^R^*, *pBR322 ori*, *amp^R^* | [^3^](#_ENREF_3) |
| pETDUET-1 | protein expression vector | Novagen |
| pBU4 | source of *repU: tet^R^* | [^16^](#_ENREF_16) |
| pIJ773 | source of *apra*^R^ | [^4^](#_ENREF_4) |
| pIJ790 | λ-Red (*gam, bet, exo*), *cat*, *araC*, *rep101*ts | [^4^](#_ENREF_4) |
| pUB307 | self-transmissible plasmid that mobilizes other plasmids in trans for DNA transfer into hosts: RP4, *neo* | [^7^](#_ENREF_7) |
| pCAP01 | gene cluster capture vector: ARSH4/CEN6, *pUC ori*, *aph(3)II*, *φC31 int-attP*, *oriT* (*RP4*). | [^2^](#_ENREF_2) |
| pCAPB1 | gene cluster capture vector: pCAP01 containing *tet^R^* and *repU* instead of *φC31 int-attP* | this study |
| pCAPB2 | gene cluster capture vector: pDR111 containing ARSH4/CEN6 | this study |
| pCAPE | gene cluster expression vector: pETDuet-1 containing sfp | this study |
| pCAPB2 -*ami* | pCAPB2 derivative that carries 47.4-kb genomic region containing the entire *ami* gene cluster (*amiA-O*). | this study |
| pCAPB2 -*ami*(ΔA) | pCAPB2-*ami* derivative (Δa*miA*): *apra^R^* | this study |
| pCAPB2 -*ami*(ΔB) | pCAPB2-*ami* derivative (Δa*miB*): *apra^R^* | this study |
| pCAPB1 -*ami* | pCAPB1 derivative that carries 47.4-kb genomic region containing the entire *ami* gene cluster (*amiA-O*). | this study |
| pCAPE -*ami* | pCAPE derivative that carries 47.4-kb genomic region containing the entire ami gene cluster (amiA-O). | this study |
| pCAPB1 -*srf* | pCAPB1 derivative that carries 38.0-kb genomic region containing the entire srf gene cluster ( srfA-D, orf1-4, sfp). | this study |
| pCAPB2 -*srf* | pCAPB2 derivative that carries 38.0-kb genomic region containing the entire srf gene cluster ( srfA-D, orf1-4, sfp). | this study |

Table S2. Oligonucleotides used in this work. Restriction sites are marked in bold; complementary sequences used for overlap extension PCR are in lower case; and underlined letters represent homology arms for recombination.

| **Gene** | **Oligonucleotide** | **(5'-3') sequence** |
| --- | --- | --- |
| ARSH4  /CEN6 | Yeast-element-NcoI-F | CTCG**CCATGG**TGTATTTAGAAAAATAAACAAATAGG |
|  | Yeast-element-SpeI-R | CTCG**ACTAGT**GTTCACGTAGTGGGCCATCG |
| *tet*^R^+*repU* | Tet-SphI-F | TCGAAC**GCATGC**GGAACGTACAGACGGCTT |
|  | Tet-XhoI-R | TCGAAC**CTCGAG**GTTACTAGTTCATCACCG |
| *sfp* | PPtase-NdeI-F | CCTG**CATATG**GCAGACGGAGGATCTAGAAT |
|  | PPtase-XhoI-R | CCTG**GGTACC**GTCAAGCTGCTGCTGAGCCG |
| *ami*-*orf*1 | ami1-Up-XhoI-F | CCGAACT**CTCGAG**ACACAGGTGTTGTAGGGACTGC |
|  | ami1-Up-BamHI-R | cagcctc**GGATCC**AAGCTCAAgAACAGTCAGCATTCTG |
| *ami*-*orf*2 | ami1-Down-BamHI-F | cttgagctt**GGATCC**GAGGCTgGCCGTAGTAGCCCA |
|  | ami1-Down-Kpn-F | TCTTTAT**GGTACC**AGTCCAGAATTGATGGCACACGA |
| *ami*-*orf*1 | ami2-up-SalI-KspI-NotI-F | CTCG**GTCGACCCGCGGAGCGGCCGC**ATGAAAAATAAATCCTTTTA |
|  | ami2-Up-Bs-NheI-R | cctggc**GCTAGC**CACCCAGCCAATCAGTAAGGC |
| *ami*-*orf*2 | ami2-Down-Bs-NheI-F | gggtg**GCTAGC**GCCAGGCCTGTTGTAATCCAG |
|  | ami2-down-SphI-ClaI-NotI-R | CTCG**GCATGCATCGATGCGGCCGC**TGGATGCTGATGGGTGTTCC |
| *amiA* | ami-Up-E-NcoI-F | CTCG**CCATGG**TGAATGGTAACTTGAA |
|  | ami-Up-E-NheI-R | gcctggc**GCTAGC**GGAAGAGCTGGTCGTATCCC |
| *ami*-*orf*2 | ami-Down-E-NheI-F | ctcttcc**GCTAGC**GCCAGGCCTGTTGTAATCCAG |
|  | ami-Down-E-EcoRI-R | CTCG**GAATTC**TGGATGCTGATGGGTGTTCC |
| *amiK* | ami-check-F | CTCATCAGGCTGCGCTGACC |
|  | ami-check-R | TTGTCAGCACATGCGCTGAGG |
| *srfAA* | srf1-Up-SpeI-F | CCGAACT**ACTAGT**CCTCATGCCTATTCTTGAAGCCA |
|  | srf1-Up-BamHI-R | ctgcgcg**GGATCC**AGGTTTCTTCGTTTCCTCCCGGC |
| *srf*-*orf6* | srf1-Down-BamHI-F | gaaacct**GGATCC**CGCGCAgCCAGCAATCTTGG |
|  | srf1-Down-KpnR | TCTTTAT**GGTACC**AGTAGCCGAGTCCGTGCGGT |
| srfA*A* | srf2-Up-SalI-F | CCTGGTCGACCCTCATGCCTATTCTTGAAGCCA |
|  | srf2-Up-NheI-R | ctgcgcgGCTAGCAGGTTTCTTCGTTTCCTCCCGGC |
| *srf*-*orf*6 | srf2-Down-NheI-F | gaaacctGCTAGCCGCGCAgCCAGCAATCTTGG |
|  | srf2-Down-NotI-R | TTATGCGGCCGCAGTAGCCGAGTCCGTGCGGT |
| *srfAB* | srf-check-F | ATGCTGAATGCGGCACGGCT |
|  | srf-check-R | GGAAGCGGCGGTCATTGCCT |
| *apra^R^* | amiA-Apra-F | ggatatgttgaatggtaacttgaatttatttcctaccaGGTGCTCACGGTAACTGATGCC |
|  | amiA-Apra-R | tccaattcttcaattgataatgaggcggtttcgACCTGGTGGAACTTATGAGCTCAGCCA |
| *apra^R^* | amiB-Apra-F | cttgttcaaatgatgagaaacgtttggctggttAccaGGTGCTCACGGTAACTGATGCC |
|  | amiB-Apra-R | atttcatgcgtctcactccttcttgcggcacggACCTGGTGGAACTTATGAGCTCAGCCA |
| *amiB* | amiB-HetEx-NheI-F | CTCG**GCTAGC**CCGCCTCATTATCAATTGAAG |
|  | amiB-HetEx-SphI-R | CTCG**GCATGC**CGGCTGAATATCAGGGATGG |

1. **Predicted functions of the *ami* genes (Table S3)**

Table S3. Proteins encoded by the *ami* cluster and open reading frames adjacent to the *ami* cluster as well as their proposed function and size. [a] AmiA-O shows very high similarity to BSI_3021- 3007 of *Bacillus subtilis* subsp. *inaquosorum* KCTC 13429 [^17^](#_ENREF_17). [b]AmiEFHG shows high similarity to ZmaGNDE, which might be responsible for hydroxymalonyl-ACP formation in the biosynthesis of zwittermicin A .

**
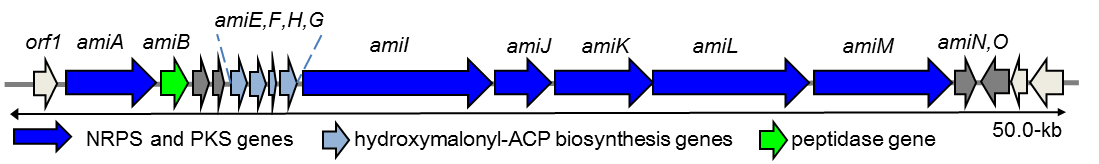
**

| Protein | Size (aa) | Proposed function | Identity/  Similarity (%) | | | Accession No |
| --- | --- | --- | --- | --- | --- | --- |
| Orf 1 | 398 | Putative MFS family major facilitator transporter[*B. subtilis*] | | 99^a^/99 | | WP_003240131.1 |
| AmiA | 1498 | Amino acid adenylation domain-containing protein [*B. subtilis*] | | 99/99 | | WP_003240128.1 |
| AmiB | 502 | β-lactamase [*B. subtilis*] | | 98/98 | | WP_003240126.1 |
| AmiC | 328 | Hypothetical protein [*B. subtilis*] | | 98/98 | | WP_003240124.1| |
| AmiD | 234 | Thioesterase [*B. subtilis*] | | 97/98 | | WP_003240123.1 |
| AmiE^b^ | 285 | 3-hydroxybutyryl-coa dehydrogenase  [*B. subtilis*] | | 99/99 | | WP_003240121.1 |
| AmiF^b^ | 354 | Methoxymalonyl-ACP biosynthesis protein  [*B. subtilis*] | | 99/99 | | WP_003240119.1 |
| AmiG^b^ | 90 | Phosphopantetheine-binding protein  [*Bacillus.* sp. JS] | | | 99/99 | WP_003240119.1 |
| AmiH^b^ | 381 | Acyl-CoA dehydrogenase (NADP(+))  [*B. subtilis*] | | 99/99 | | WP_003240115.1 |
| AmiI | 3032 | Nonribosomal peptide synthetase-polyketide synthase hybrid [*B. subtilis*] | | 98/98 | | WP_003240114.1 |
| AmiJ | 890 | Nonribosomal peptide synthetase subunit  [*B. subtilis*] | | 98/98 | | WP_003240112.1 |
| AmiK | 1509 | Putative polyketide synthase PksJ (PKS)  [*B. subtilis*] | | 98/98 | | WP_003240111.1 |
| AmiL | 2518 | Polyketide synthase subunit [*B. subtilis*] | | 98/98 | | WP_003240108.1 |
| AmiM | 2143 | Polyketide synthase subunit [*B. subtilis*] | | 98/98 | | WP_003240106.1 |
| AmiN | 334 | Putative kinase [*B. subtilis*] | | 99/99 | | WP_003240104.1 |
| AmiO | 459 | Alkaline phosphatase [*B. subtilis*] | | 99/99 | | WP_003240102.1 |
| Orf 2 | 231 | Membrane component [*B. subtilis*] | | 99/99 | | WP_003240098.1 |

1. **Chemical structures of selected natural products from the genus *Bacillus* (Figure S1)**

Figure S1. Chemical structures of selected natural products from the genus *Bacillus*.

**5. Physical maps of pCAPB1, pCAPB2, pCAPE, and their *srf* specific derivatives.**


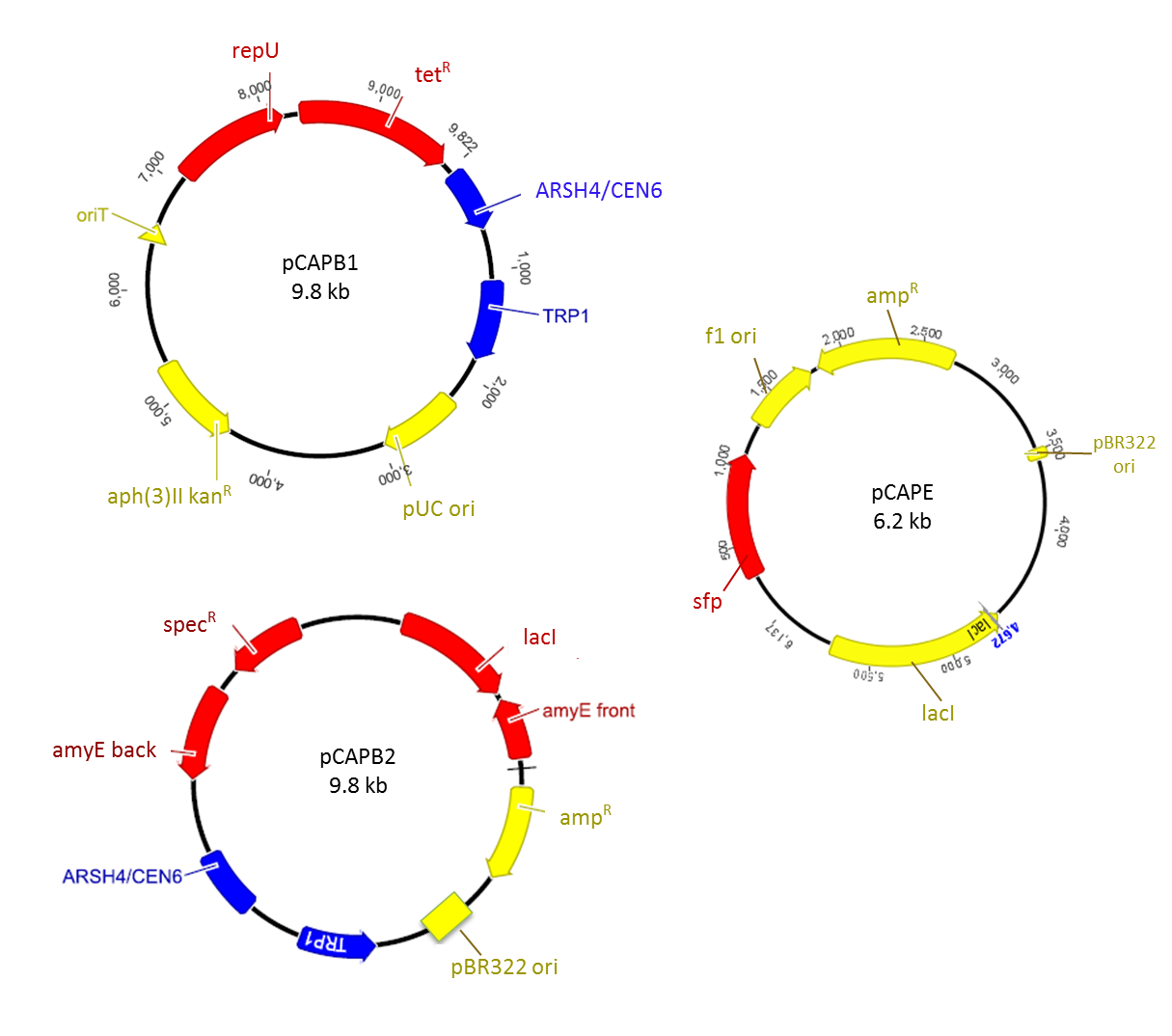


Figure S2. Physical maps of the capture vectors for TAR direct cloning and heterologous expression. The vector pCAPB01 consists of three elements, including the yeast element of ARSH4/CEN6 (replication origin) and TRP1 auxotrophic marker, the *E. coli* element of an ampicillin resistance gene (*amp^R^*) and the *Bacillus* elements of DNA sequence for integration into the *B. subtilis amyE* gene, the *lac* repressor *lacI* and a spectinomycin resistance gene (*spec^R^*). The pCAPE vector was generated from commercial vector pETDuet-1 with the insertion of the phosphopantetheine transferase (PPTase) gene *sfp* in MCS2.

A.


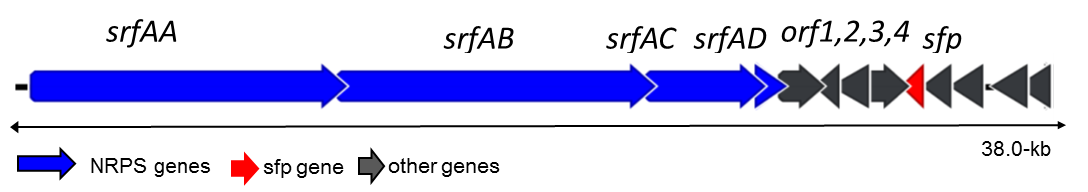


B.


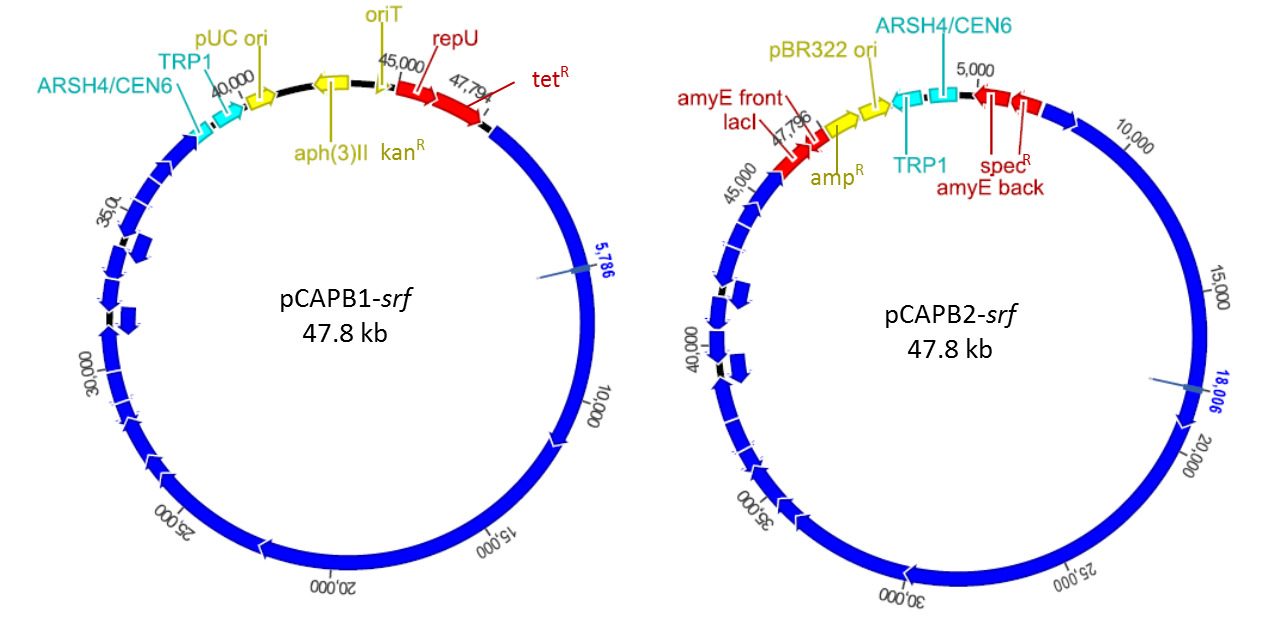


Figure S3. (A) Organization of the *srf* biosynthetic gene cluster in *B. subtilis* 1779. (B) Physical maps of the TAR-cloned *srf* gene cluster. The 38-kb genomic region containing the *srf* gene cluster was directly cloned in yeast, yielding pCAPB1-*srf* and pCAPB2-*srf*.

**6. Results of heterologous expression of the *srf* gene cluster in *B. subtilis* JH642 ROM77 (Figure S4)**


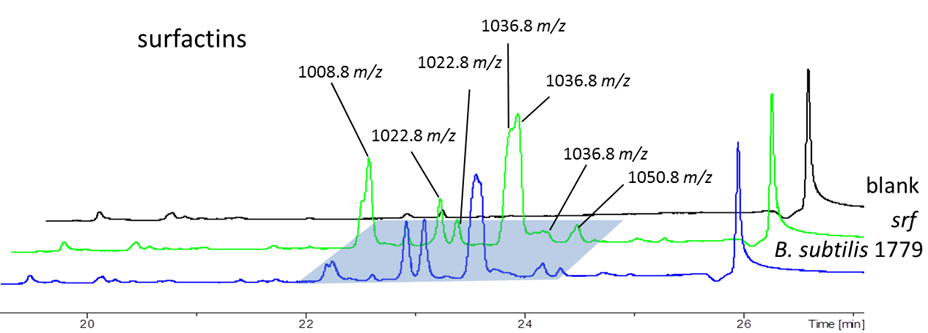


Figure S4. UPLC-MS analyses of heterologously produced surfactins*.* LCMS UV traces represent the relative production of surfactins in the native *B. subtilis* 1779 and *B. subtilis* ROM77 carrying pCAPB2 (blank) and pCAPB2-*srf*. Detection was at 210 nm.

**7. Restriction mappings of pCAPB1-*ami*, pCAPB2-*ami*, and pCAPE-*ami* (Figure S5).**


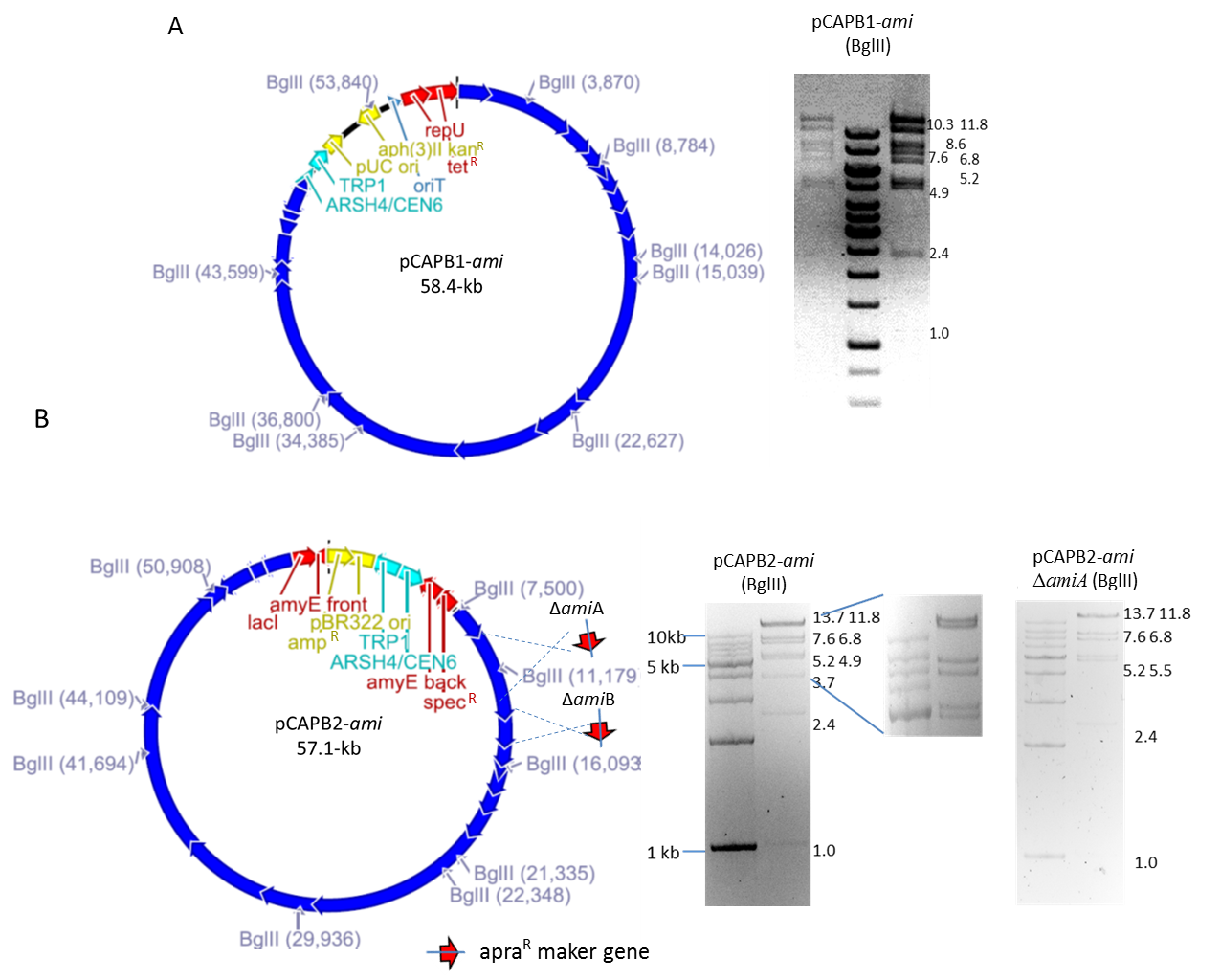


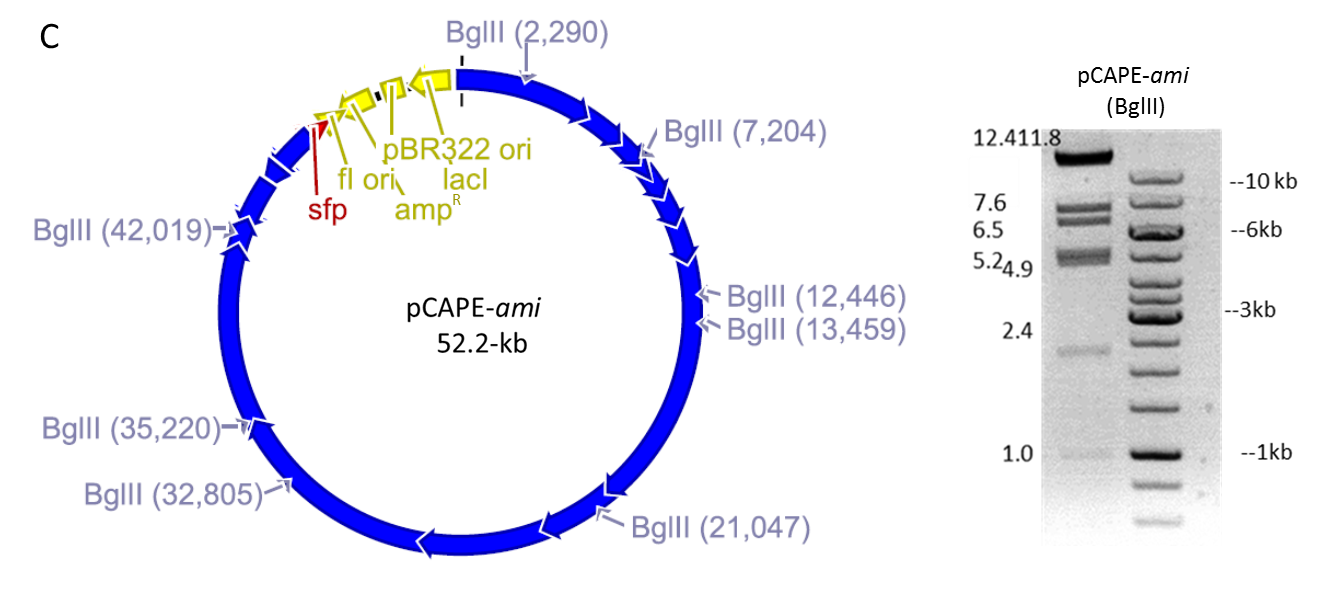


Figure S5. Physical maps of the TAR-cloned *ami* gene cluster and its derivatives. The 47.4-kb genomic region containing the *ami* gene cluster was directly cloned in yeast, resulting in pCAPB1-*ami* (A) and pCAPB2-*ami* (B). The *amiA* and *amiB* were replaced with the *apra^R^* antibiotic marker gene *via* λ-Red recombination mediated PCR targeting in *E. coli*, resulting in mutated constructs of pCAPB2-*ami* (Δ*amiA*, Δ*amiB*). Successful gene deletions were confirmed by BglII restriction mapping, as shown in gel picture on right (B). The pCAPB1 backbone and *orf*1 on pCAPB1-*ami* were replaced with the pCAPE backbone *via* λ-Red mediated recombination in *E. coli*, generating pCAPE-*ami* for expression in *E. coli* (C).

**8. Results of heterologous expression of the *ami* gene cluster in *E. coli* BL21(DE3) (Figure S6)**


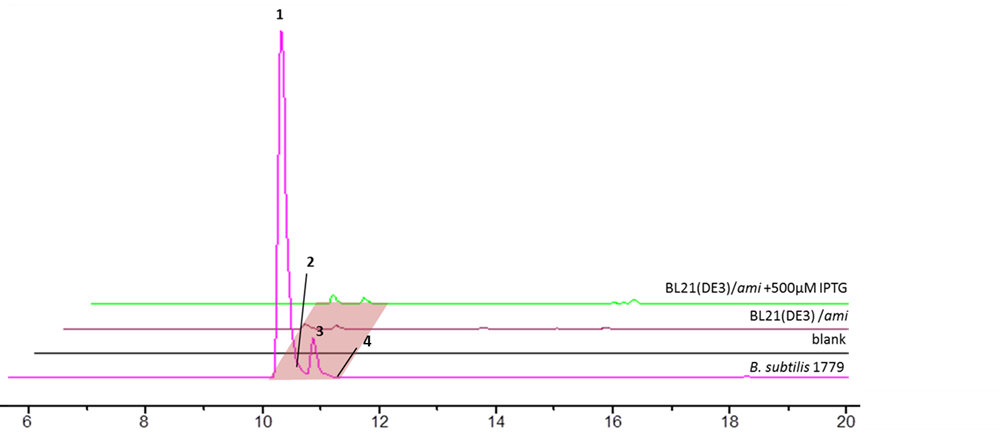


Figure S6. LCMS extracted ion chromatogram of amicoumacins (**1**-**6**) produced by *E. coli* BL21(DE3) carrying pCAPE-*ami* (0, 500 μM IPTG), the empty vector pCAPE (blank), and wild type producer *B. subtilis* 1779. All chromatograms were scaled at the same intensity.

**9. Results of feeding experiments for biosynthetic pathway study (Table S4)**

Table S4. Results of feeding experiments for biosynthetic pathway study

| compound | Feeding  experiment | *m/z*  [M + H]^+^ | Mol. formula | BPF^a^ *m/z*  [M+H]^+^ | | Sum formula |
| --- | --- | --- | --- | --- | --- | --- |
| amicoumacin A |  | 424.21 | C_20_H_30_N_3_O_7_ | | 250.14 | C_14_H_20_NO_3_ |
|  | ^15^N-Asn | 426.20 | C_20_H_30_N^15^N_2_O_7_ | | 250.14 | C_14_H_20_NO_3_ |
|  | TFL^b^ | 478.17 | C_20_H_27_F_3_N_3_O_7_ | | 304.12 | C_14_H_17_F_3_NO_3_ |
| amicoumacin B |  | 425.20 | C_20_H_29_N_2_O_8_ | | 250.14 | C_14_H_20_NO_3_ |
|  | ^15^N-Asn | 426.19 | C_20_H_29_N^15^NO_8_ | | 250.14 | C_14_H_20_NO_3_ |
|  | TFL | 479.15 | C_20_H_26_F_3_N_2_O_6_ | | 304.11 | C_14_H_17_F_3_NO_3_ |
| amicoumacin C |  | 407.18 | C_20_H_27_N_2_O_7_ | | 250.14 | C_14_H_20_NO_3_ |
|  | ^15^N-Asn | 408.17 | C_20_H_27_N_15_NO_7_ | | 250.14 | C_14_H_20_NO_3_ |
|  | TFL | 461.14 | C_20_H_24_F_3_N_2_O_7_ | | 304.11 | C_14_H_17_F_3_NO_3_ |

a, BPF (benzopyran-1-one fragment) b, TFL=5,5,5-Trifluoro-leucine

**10. NMR and MS analysis (Table S5, Figures S7, and S8)**

**Table S5. NMR data of preamicoumacins A (5) (*d_6_*-DMSO)**

|  | | **5** | | | | | | | |
| --- | --- | --- | --- | --- | --- | --- | --- | --- | --- |
| position | | *δ*_C_, mult | | | | *δ*_H_, (*J* in Hz) | | | HMBC |
| 1 | | 169.5, C | | | |  | | |  |
| 3 | | 81.5, CH | | | | 4.67, dt (13.0, 2.5) | | | 1 |
| 4 | | 29.1, CH_2_ | | | | 2.85, d (16.0), 3.08, dd(2.5,16.0) | | | 5, 9, 10 |
| 5 | | 115.6, CH | | | | 6.81, d (7.8) | | | 4, 7, 9 |
| 6 | | 136.7, CH | | | | 7.47, dd (7.5, 7.8) | | | 8, 10 |
| 7 | | 118.9, CH | | | | 6.85, d (7.5) | | | 5, 8, 9, |
| 8 | | 161.3, C | | | |  | | |  |
| 9 | | 108.7, C | | | |  | | |  |
| 10 | | 141.2, C | | | |  | | |  |
| 1' | | 22.0, CH_3_ | | | | 0.89, d ( 6.6) | | | 2', 4' |
| 2' | | 23.7, CH_3_ | | | | 0.97, d ( 6.6) | | | 1', 4' |
| 3' | | 24.5, CH | | | | 1.68, m | | |  |
| 4' | | 39.1, CH_2_ | | | | 1.43, m, 1.82, m | | |  |
| 5' | | 48.6, CH | | | | 4.20, m | | | 7' |
| 6'-N | | NH | | | | 7.97, d (8.9)  7.90 d (9.1) | | | 7' |
| 7' | | 172.6, C | | | |  | | |  |
| 8' | | 71.8, CH | | | | 3.87, dd (6.5, 6.0) | | | 7', 10' |
| 8'-OH | |  | | | | 5.18, d (6.5) | | | 7', |
| 9' | | 74.5, CH | | | | 3.56, dd (6.0, 10.5) | | | 7', 12' |
| 9'-OH | |  | | | | 5.23, d (6.0) | | | 11' |
| 10' | | 48.0, CH | | | | 4.20, m | | | 14' |
| 10'-NH | | NH | | | | 7.80, d (9.1) | | | Asn-1 |
| 11' | | 35.4, CH_2_ | | | | 2.29, m, 2.32, m | | | 12' |
| 12' | | 173.5, C | | | |  | | |  |
| Asparagine (Asn) | | | | |  |  | | |  |
| 1 | | | 171.5, C | | |  | | |  |
| 2 | | 50.2, CH | | | | 4.47, m | | Asn-1, 4, FA-1 | |
| 2-NH | | NH | | | | 7.92, d (8.0) | | FA-1 | |
| 3 | | 37.8, CH_2_ | | | | 2.36, m, 2.44, dd (7.5, 15.5) | | | Asn-1, 5 |
| 4 | | 171.9, C | | | |  | | |  |
| Fatty acid (FA) | | | |  | |  | | |  |
| 1 | 172.6, C | | | | |  | | |  |
| 2 | | 35.6, CH_2_ | | | | 2.07, t (7.5) | | | FA-1 |
| 3 | | 27.3, CH_2_ | | | | 1.47, m | | |  |
| 4~8 | | 30.5-31.2, CH_2_ | | | | | 1.18-1.21, m | |  |
| 9 | | 38.9, CH_2_ | | | | 1.22, m | | |  |
| 10 | | 27.6, CH | | | | 1.45, m | | |  |
| 11 | | 23.0, CH_3_ | | | | 0.83, d (0.75) | | | FA-9 |
| 12 | | 23.0, CH_3_ | | | | 0.83, d (0.75) | | | FA-9 |

|  | Figure S7. Selected HMBC and COSY correlations of preamicoumacin A (**5**).    Figure S8. ESI-MS fragmentations of preamicoumacins A-B (**5**-**6**). |
| --- | --- |
|  | **11. Antibacterial bioactivity of preamicoumacins (Table S6)**  Table S6. Antibacterial activities of amicoumacins against their native producer *B. subtilis* 1779 and *Staphylococcus aureus* UST950701-005.   \| Antibacterial activities (MIC, µg ml^-1^) \| \| \|  \| \| --- \| --- \| --- \| --- \| \| amicoumacins \| *B. subtilis* 1779 \| *S. aureus* UST950701-005 \| \| \| amioucoumacin A (**1**) \| 20 \| 5 \| \| \| amioucoumacin B **2** \| >100 \| >100 \| \| \| amioucoumacin C **3** \| >100 \| >100 \| \| \| *O*-methylamioucoumacin B (**4)** \| >100 \| >100 \| \| \| preamicoumacin A **(5)** \| >100 \| >100 \| \| \| preamicoumacin B **(6)** \| >100 \| >100 \| \|   **12. Efficiencies of conjugation between *E. coli* and *Bacillus* (Table S7)**  Table S7. Transformation efficiency of three constructs (pCAPB1, pCAPB1-*ami* and pCAPB1-*srf*) into 5 *Bacillus* hosts via conjugation.   \| Expression host \| pCAPB1 \| pCAPB1-*srf* \| pCAPB1-*ami* \| \| --- \| --- \| --- \| --- \| \| Transformatants (cells/mL) \| Transformatants (cells/mL) \| Transformatants (cells/mL) \| \| *B. subtilis*168 \| 100**-**200 \| 10-20 \| 10-20 \| \| *B. subtilis* ROM77 \| 50-200 \| 5-10 \| 10-20 \| \| *B. subtilis* JH642+*sfp* \| 200-500 \| 20-50 \| 20-50 \| \| *B. thuringiensis* GBJ001 \| 500-2000 \| 10-50 \| 20-50 \| \| *B. thuringiensis* BMB171 \| 200-500 \| 10-20 \| 5-10 \| |

**13. References**

1. Kouprina, N. & Larionov, V. Selective isolation of genomic loci from complex genomes by transformation-associated recombination cloning in the yeast *Saccharomyces cerevisiae*. *Nat. Protoc.* **3**, 371-377 (2008).

2. Yamanaka, K.*, et al.* Direct cloning and refactoring of a silent lipopeptide biosynthetic gene cluster yields the antibiotic taromycin A. *Proc. Natl. Acad. Sci. USA* **111**, 1957-1962 (2014).

3. Wagner, J. K., Marquis, K. A. & Rudner, D. Z. Sira enforces diploidy by inhibiting the replication initiator dnaa during spore formation in *Bacillus subtilis*. *Mol. Microbiol.* **73**, 963-974 (2009).

4. Gust, B.*, et al.* Pcr-targeted streptomyces gene replacement identifies a protein domain needed for biosynthesis of the sesquiterpene soil odor geosmin. *Proc. Natl. Acad. Sci. USA* **100**, 1541-1546 (2003).

5. Jensen, G. B.*, et al.* The genetic basis of the aggregation system in *Bacillus thuringiensis* subsp. *Israelensis* is located on the large conjugative plasmid PXO16. *J. Bacteriol.* **177**, 2914-2917 (1995).

6. He, J.*, et al.* Complete genome sequence of *Bacillus thuringiensis* mutant strain BMB171. *J. Bacteriol.* **192**, 4074-4075 (2010).

7. Flett, F., Mersinias, V. & Smith, C. P. High efficiency intergeneric conjugal transfer of plasmid DNA from *Escherichia coli* to methyl DNA-restricting *Streptomycetes*. *FEMS Microbiol Lett.* **155**, 223-229 (1997).

8. Albano, M., Hahn, J. & Dubnau, D. Expression of competence genes in *Bacillus subtilis. C***169**, 3110-3117 (1987).

9. Itoh, J.*, et al.* Amicoumacin-A, a new antibiotic with strong anti-inflammatory and antiulcer activity. *J Antibiot (Tokyo)* **34**, 611-613 (1981).

10. Li, Y. X.*, et al.* Five new amicoumacins isolated from a marine-derived bacterium *Bacillus subtilis.* *Mar. Drugs* **10**, 319-328 (2012).

11. Fujii, K.*, et al.* A nonempirical method using lc/ms for determination of the absolute configuration of constituent amino acids in a peptide: Combination of Marfey's method with mass spectrometry and its practical application. *Anal. Chem.* **69**, 5146-5151 (1997).

12. Fujii, K.*, et al.* Further application of advanced Marfey's method for determination of absolute configuration of primary amino compound. *Tetrahedron Lett.* **39**, 2579-2582 (1998).

13. Donnellan, J. E., Jr., Nags, E. H. & Levinson, H. S. Chemically defined, synthetic media for sporulation and for germination and growth of *Bacillus subtilis*. *J. Bacteriol.* **87**, 332-336 (1964).

14. Solomon, J. M., Magnuson, R., Srivastava, A. & Grossman, A. D. Convergent sensing pathways mediate response to two extracellular competence factors in *Bacillus subtilis*. *Genes Dev.* **9**, 547-558 (1995).

15. Nakano, M. M., Marahiel, M. A. & Zuber, P. Identification of a genetic-locus required for biosynthesis of the lipopeptide antibiotic surfactin in *Bacillus subtilis*. *J. Bacteriol.* **170**, 5662-5668 (1988).

16. Bourgouin, C., Delecluse, A., Delatorre, F. & Szulmajster, J. Transfer of the toxin protein genes of bacillus-sphaericus into *Bacillus thuringiensis* subsp *israelensis* and their expression. *Appl. Environ. Microbiol.* **56**, 340-344 (1990).

17. Yi, H., Chun, J. & Cha, C. J. Genomic insights into the taxonomic status of the three subspecies of *Bacillus subtilis*. *Syst. Appl. Microbiol.* **37**, 95-99 (2014).

18. Chan, Y. A.*, et al.* Hydroxymalonyl-acyl carrier protein (ACP) and aminomalonyl-ACP are two additional type I polyketide synthase extender units. *Proc. Natl. Acad. Sci. USA* **103**, 14349-14354 (2006).

19. Reimer, D., Luxenburger, E., Brachmann, A. O. & Bode, H. B. A new type of pyrrolidine biosynthesis is involved in the late steps of xenocoumacin production in *Xenorhabdus nematophila*. *Chembiochem* **10**, 1997-2001 (2009).

**14.** ESI-MS analysis of preamicoumacins A-B (**5-6**)


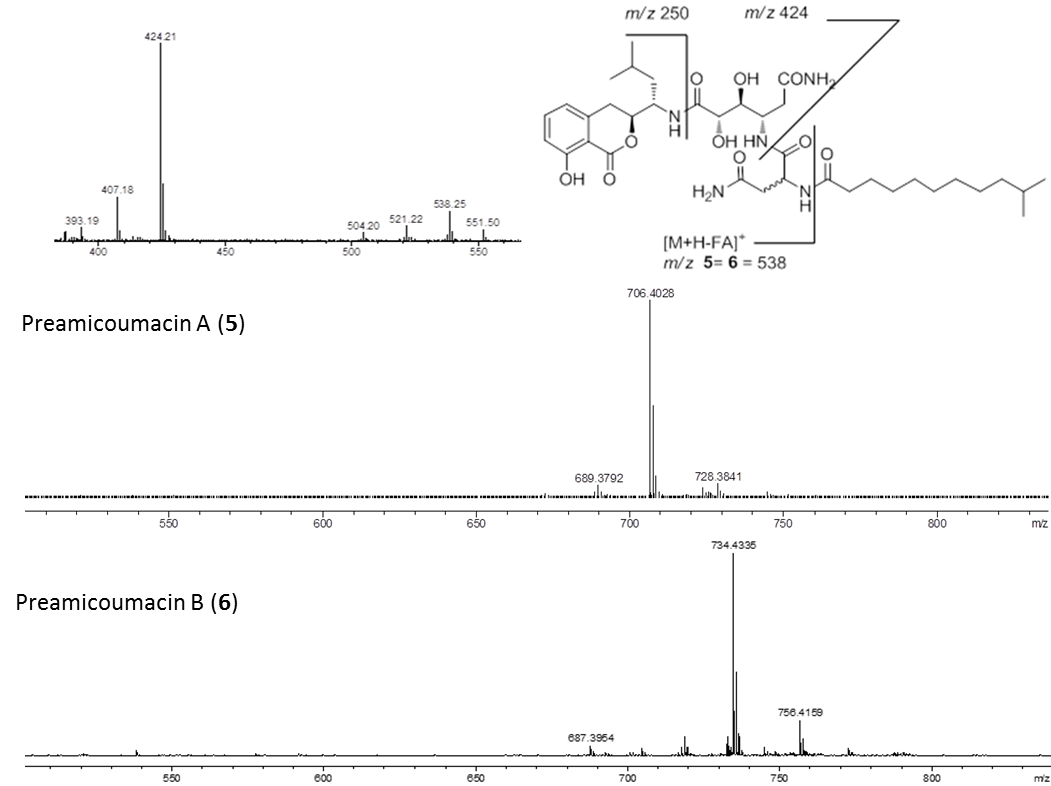


^1^HNMR of preamicoumacin A (DMSO-*d_6_*, 500MHz)

^13^C-NMR of preamicoumacin A (**5**) (DMSO-*d_6_*, 125MHz)

^1^H-^1^H COSY of preamicoumacin A (**5**) (DMSO-*d_6_*, 500MHz)

^1^H -^13^C HSQC of preamicoumacin A (**5**) (DMSO-*d_6_*, 500MHz)

^1^H -^13^C HMBC of preamicoumacin A (**5**) (DMSO-*d_6_*, 500MHz)
